# Reconstructing 3D deformation dynamics for curved epithelial sheet morphogenesis from positional data of sparsely-labeled cells

**DOI:** 10.1038/s41467-017-00023-7

**Published:** 2017-05-02

**Authors:** Yoshihiro Morishita, Ken-ichi Hironaka, Sang-Woo Lee, Takashi Jin, Daisuke Ohtsuka

**Affiliations:** 1 Laboratory for Developmental Morphogeometry, RIKEN Quantitative Biology Center, Kobe, 650-0047 Japan; 2 Research Fellow of the Japan Society for the Promotion of Science, Tokyo, Japan; 3 Laboratory for Nano-Bio Probes, RIKEN Quantitative Biology Center, Osaka, 565-0874 Japan

## Abstract

Quantifying global tissue deformation patterns is essential for understanding how organ-specific morphology is generated during development and regeneration. However, due to imaging difficulties and complex morphology, little is known about deformation dynamics for most vertebrate organs such as the brain and heart. To better understand these dynamics, we propose a method to precisely reconstruct global deformation patterns for three-dimensional morphogenesis of curved epithelial sheets using positional data from labeled cells representing only 1–10% of the entire tissue with limited resolution. By combining differential-geometrical and Bayesian frameworks, the method is applicable to any morphology described with arbitrary coordinates, and ensures the feasibility of analyzing many vertebrate organs. Application to data from chick forebrain morphogenesis demonstrates that our method provides not only a quantitative description of tissue deformation dynamics but also predictions of the mechanisms that determine organ-specific morphology, which could form the basis for the multi-scale understanding of organ morphogenesis.

## Abstract (web summary)

Quantifying deformation patterns of curved epithelial sheets is challenging owing to imaging difficulties. Here the authors develop a method to obtain a quantitative description of 3D tissue deformation dynamics from a small set of cell positional data and applied it to chick forebrain morphogenesis.

## Introduction

Understanding how three-dimensional (3D) organ morphology is determined during development and regeneration is one of the ultimate goals in biology. This is important not only for pure scientific interests but also for potential medical applications for controlling and designing functional organs. To achieve these goals, it is essential to clarify the quantitative relationships between microscopic molecular/cellular activities and organ-level tissue deformation dynamics^1^. While the former have been studied for several decades, the latter—macroscopic geometrical information about physical tissue deformation—has been lacking. Recent advancements in imaging techniques and fluorescent probes have made total cell recordings possible, especially in flat, small, and relatively transparent tissues such as Drosophila germband and wing, and Zebrafish skin^2–6^. Based on tracking data, collective cellular behaviors and tissue deformation dynamics at single-cell resolution during development have been studied using velocimetric methods.

Aside from these notable exceptions, we have little knowledge of tissue deformation dynamics during the morphogenesis of many vertebrate organs including brain, heart, and even artificially synthesized organoids derived from ES and iPS cells^7–10^. One reason for this lack of information is the difficulty in measurement; in general, tissue morphologies are achieved through complex 3D deformation of highly curved sheet-like structures, either cystic or tubular. Furthermore, high resolution deep imaging is often quite difficult. In addition, to achieve high temporal resolution, methods of embryo culture which maintain normal function over an appropriate period are required. From the analytical perspective, image processing that can automatically distinguish individual cell trajectories from a dense cell population is often difficult, which itself is an important issue in this field^11, 12^. Furthermore, although sheet deformation occurs in 3D space, its actual structure is two-dimensional (2D) even with curvature. Thus, in order to analyze deformation dynamics, it is necessary to introduce a 2D curvilinear coordinate system onto the sheet with the involvement of a non-Euclidean metric. As will be described below, the 2D coordinate system and metric is, in general, different at each developmental time point with differing morphology, making it difficult to perform ordinary velocimetric analysis.

Against this background, we propose a method to reconstruct tissue deformation dynamics for 3D morphogenesis of curved epithelial sheets from a small set of positional cellular data with limited resolution. This method is a generalization of that proposed in our previous study which focused on flat tissues^13^. By combining differential-geometrical and Bayesian frameworks, the difficulties listed above are overcome. In particular, with this method, manifold- and tensor-based descriptions are adopted, allowing it to be applied to any tissue described with arbitrary coordinate systems. This is critically important for analyzing the deformation of curved structures because orthonormal coordinate systems cannot be applied to them and because curvilinear coordinate systems defining the surface itself can differ with changes in morphology. With our method, positional information from just 1–10% of the total cells within a tissue is adequate for reconstructing the global deformation pattern with sufficient accuracy, which ensures the feasibility of analyzing many vertebrate organs with complex morphologies. Moreover, the sparse cell labeling makes it easier to distinguish individual cells even if the microscopic resolution is not high.

**Fig. 1:**
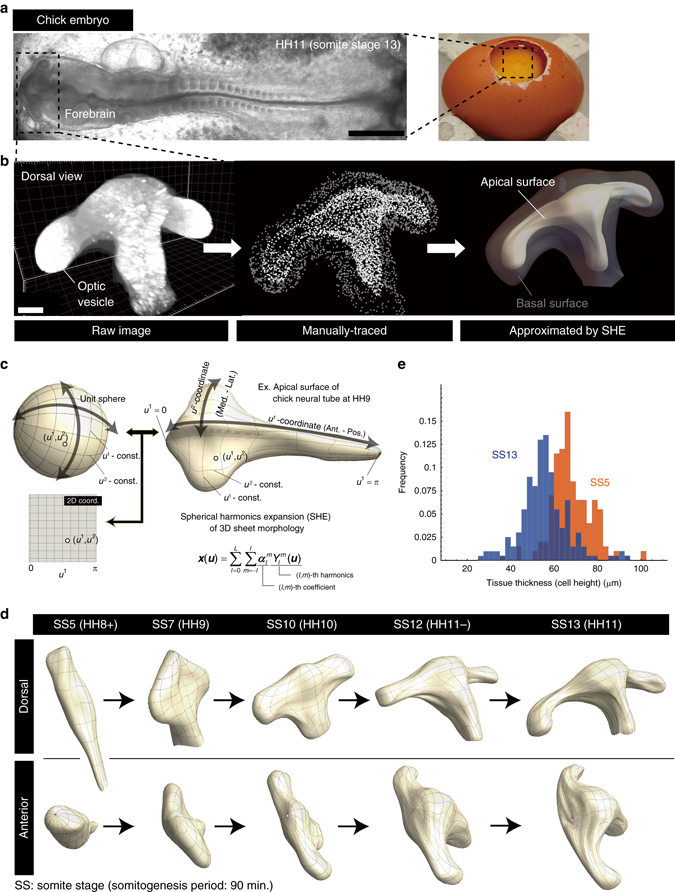
Chick forebrain formation as an example of 3D epithelial sheet morphogenesis. **a** Chick embryo at HH11 (SS13). Scale bar: 500 μm. **b** Methods for modeling forebrain 3D morphology. Using a two-photon microscopic image (*left*), apical and basal surfaces were manually traced. The traced data were represented as a set of dots for each surface. From the traced data, smooth 3D morphologies of both the apical (*white*) and basal (*gray*) surfaces were obtained using the spherical harmonics approximation (*right*). During the developmental stages studied, the forebrain has a single-layered structure. Scale bar: 100 μm. **c** Spherical harmonics expansion (SHE) of 3D sheet morphology. This expansion not only provides a smooth approximation of complex sheet morphology but also a 2D coordinate system on the sheet. Since epithelial tissues often have sac-like or tubular morphologies that can be approximated by closed surfaces, SHE is a convenient way to define the 2D curvilinear coordinates on the surface (see also Fig. 2 and Supplementary Notes). **d** Time course of the morphological changes of the apical surfaces of the forebrain from SS5 (HH8+) to SS13 (HH11). **e** Distribution of tissue thickness. From SS5 to SS13, the thickness is almost spatially uniform although the mean values gradually decrease (see Supplementary Fig. 7 for details)

The performance of the proposed method is validated using both simulated and in vivo data. In particular, we focus on the process of tissue evagination and confirm with simulated data that the spatial patterns of deformation characteristics calculated from reconstructed tissue deformation maps show clear signatures for distinguishing different mechanisms that generate similar morphologies. Then, as an actual biological target, we apply this method to morphogenesis during early development of the chick forebrain from somite stage (SS) 5 to SS13; SS5 corresponds to the very beginning of optic vesicle (OV) formation from a simple neural tube and at SS13 a fully evaginated OV and overall more complex morphology is present (Fig. 1). The tissue deformation analysis shows that globally aligned anisotropic deformation (i.e., biased tissue stretching) along the medio-lateral axis, rather than local area growth, is the predominant morphogenetic mechanism that occurs throughout the entire period of our focus (i.e., SS5-SS13). This is supported by experiments in which tissue evagination and OV elongation could still be observed even though cell proliferation has been inhibited, although overall size changes slightly. Quantification of cellular characteristics (specifically cell size, shape, and division orientation) suggests that this tissue-level anisotropic deformation is driven by cell rearrangement. Thus, our analysis not only provides quantitative descriptions of tissue deformation dynamics but also enables the prediction or narrowing of mechanisms that determine organ-specific morphology, which could form the basis for multi-scale understanding of organ morphogenesis.

## Results

### 2D coordinates on curved surfaces and metric anisotropy

Within epithelial tissues, adjacent cells are tightly connected through apical junctions^14, 15^, enabling the maintenance of sheet-like structures during morphogenesis. In the case of forebrain morphogenesis, the neuroepithelial sheet maintains a single layer of columnar cells of almost uniform height throughout each developmental stage from SS5 to SS13 (Fig. 1e and Supplementary Fig. 7C, and see also Supplementary Fig. 7A, B for methods on measuring cell height or tissue thickness). In the following, we propose a method to reconstruct the deformation dynamics of such sheet-like structures from a limited data set. We first give a brief overview of differential-geometrical descriptions of a surface and its deformation (see Supplementary Notes and textbooks such as refs 16, 17 for details), then describe how a Bayesian statistical model for inferring deformation maps is developed.

**Fig. 2:**
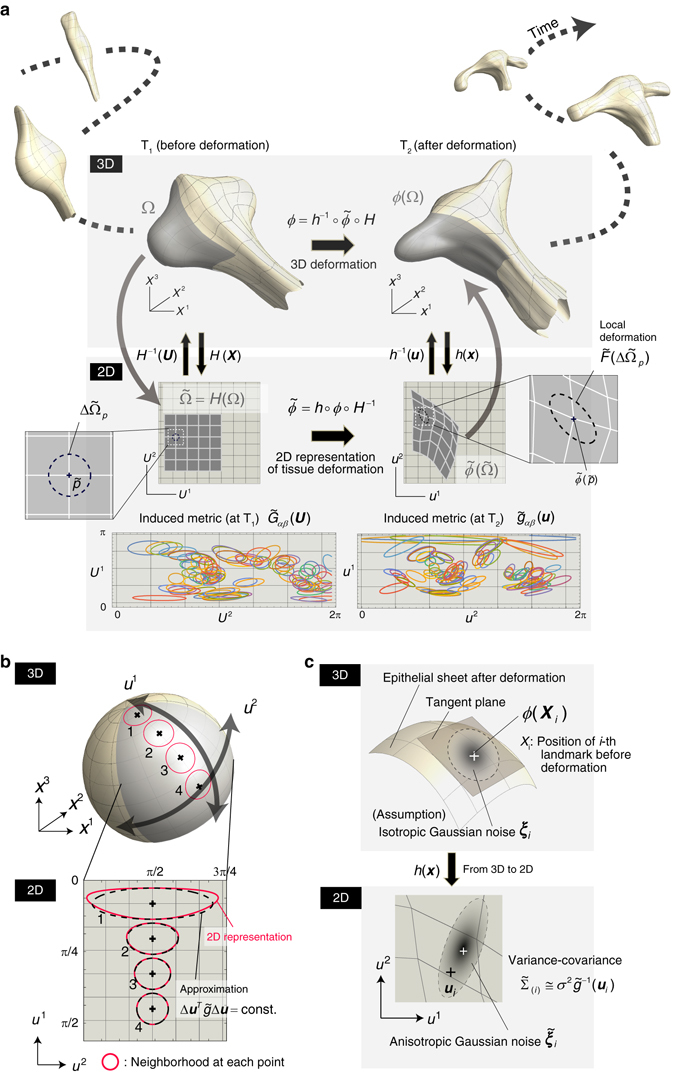
Mathematical description of epithelial-sheet deformation. **a** Geometrical view of tissue deformation. When the scale of organs is much larger than that of a cell, tissue-level deformation can be described as a map of continuum. The deformation map of a surface has 3D and 2D representations (***x*** = *ϕ*(***X***) and ${\boldsymbol{u}}=\tilde{\phi }({\boldsymbol{U}})$, respectively). *H*(***X***) and *h*(***x***) are functions that indicate how to transform a 3D coordinate into 2D at different time points (T_1_ and T_2_). With these functions, the relationship $\phi ={h}^{-1}\circ \tilde{\phi }\circ H$ holds. In our method, using positional data from sparsely labeled cells, the 2D map modeled by lattice deformation is estimated; the deformation of the gray region from $\tilde{\Omega }$ to $\tilde{\phi }(\tilde{\Omega })$ shows an example. The regions $\tilde{\Omega }$ and $\tilde{\phi }(\tilde{\Omega })$ can be linked to their 3D representations (i.e., Ω and *ϕ*(Ω)) through the functions *H* ^−1^(***U***) and *h* ^−1^(***u***), respectively. After the map is determined, local tissue deformation can be calculated from the deformation gradient tensor $\tilde{{\boldsymbol{F}}}$. In the figure, the local deformation around a point $\tilde{p}$ in a 2D coordinate system (from $\Delta {\tilde{\Omega }}_{p}$ to $\tilde{\boldsymbol{F}}(\Delta {\tilde{\Omega }}_{p})$) is shown. As a result of compressing a curved surface into a flat plane, the metric differs depending on the position in the 2D coordinate systems (${\tilde{G}}_{\alpha \beta }({\boldsymbol{U}})$ and ${\tilde{g}}_{\alpha \beta }({\boldsymbol{u}})$). The *bottom* figure shows examples of the positional dependence of metric calculated from the spherical harmonics used for modeling the 3D morphologies at time points T_1_ and T_2_ shown in the figure. Different ellipses with different colors show the metric anisotropy at their positions. **b** Using the metric $\tilde{g}$, the neighborhood of each point in the 3D representation (Δ***x*** ^*T*^Δ***x*** = const., shown as *red circles*) can be approximated by $\Delta {{\boldsymbol{u}}}^{T}\tilde{g}\Delta {\boldsymbol{u}}=$ const. in 2D. **c** In the process of estimating deformation maps from landmark positions, we assumed that the observed positions include an additive noise obeying isotropic Gaussian distribution (***ξ***_*i*_) on the tangent plane at each point in the 3D representation. In the 2D representation, the noise distribution becomes anisotropic (${\tilde{{\boldsymbol{\xi }}}}_{i}$) due to metric anisotropy. The variance-covariance matrix was modeled as ${\tilde{\Sigma }}_{(i)}\cong {\sigma }^{2}{\tilde{g}}^{-1}({{\boldsymbol{u}}}_{i})$ using the metric at the observed position in the 2D coordinate system of each landmark. *σ* represents noise magnitude. Including this noise anisotropy into the likelihood function improves the estimation performance (see Fig. 4)

In most microscopic imaging data, cell position within an epithelial sheet is given as a 3D coordinate. However, since a sheet is actually a 2D structure embedded in 3D space, and the position of each cell is generally restricted within the sheet during development (except for processes such as delamination and cell death), the deformation of a sheet can essentially be described as a 2D map that relates the positions before and after deformation for each cell. Thus, in order to analyze sheet deformation dynamics, we need to start by allotting 2D coordinates to points on a curved sheet. Epithelial tissues often have sac like or tubular morphologies that can be approximated by closed surfaces, for which the spherical harmonics expansion (SHE) (i.e., representation as a weighted sum of multiple harmonics) would be the best choice for obtaining 2D coordinate charts (Figs. 1c and 2a, and see also Supplementary Note 1 for details). This representation means a smooth mapping of a given closed surface onto a unit sphere, and intuitively the 2D coordinates on the sheet can be interpreted as a pair of the latitude and longitude. In the example of chick forebrain shown in Fig. 1c, a 2D coordinate was chosen so that its anterior (or posterior) end corresponds to the “Arctic” (or “Antarctic”) pole.

As a result of compressing a curved surface into a flat plane, the metric (roughly, a type of ruler) differs depending on the position in the given 2D coordinate system (Fig. 2a(bottom), b and Supplementary Note 1). This is intuitively understandable by considering the relationship between the surface of the earth in 3D space and a flattened 2D world atlas, for example. In this case, a circular neighborhood of the same size (small relative to the curvature radius) at each point looks very different depending on its position within the 2D atlas; this is due to the difference in the metric $\tilde{g}$ between points on the atlas (Fig. 2b). Since the deviation Δ***x*** from each point in 3D space and its correspondence in 2D Δ***u*** should have the same size, the metric, called the induced metric, satisfies the following relationship (Supplementary Note 1):

1 $${\Vert \Delta {\boldsymbol{x}}\Vert }^{2}=\Delta {{\boldsymbol{x}}}^{T}\Delta {\boldsymbol{x}}=\Delta {{\boldsymbol{u}}}^{T}\tilde{g}\Delta {\boldsymbol{u}}={\Vert \Delta {\boldsymbol{u}}\Vert }^{2}.$$

This metric $\tilde{g}$ plays key roles in developing statistical models for the inference of tissue deformation maps and in calculating spatiotemporal patterns of tissue deformation characteristics after the map is determined.

### Geometrical characterization of tissue deformation dynamics

**Fig. 3:**
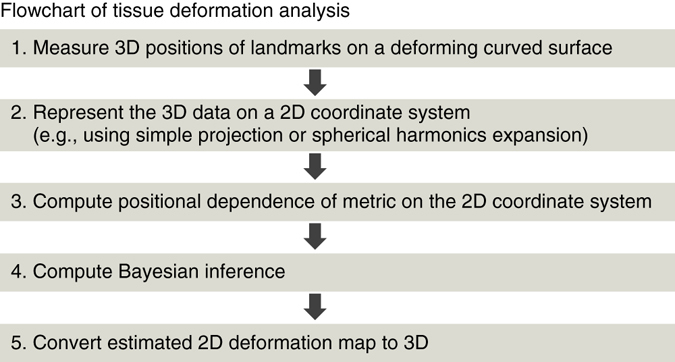
Flowchart of estimating tissue deformation maps from landmark positions

Organ-specific morphology is achieved through complex tissue deformation dynamics resulting from cellular processes such as division, apoptosis, and intercellular rearrangement^18^. As is the case in many vertebrate organs, the scale of the whole organ (10^2^−10^4^ μm) is generally much larger than that of a single cell (10^0^−10^1^ μm), and thus it is useful in tissue-level deformation analysis to regard them as continuums by averaging the variability in the shape and size of individual cells. Continuum deformation can be described as a map, and as with positional coordinates of cells, the deformation map also has 3D (denoted by ***x*** = *ϕ*(***X***)) and 2D (${\boldsymbol{u}}=\tilde{\phi }({\boldsymbol{U}})$) representations (Fig. 2a). For a given pair of 2D coordinate charts before and after deformation (*H* and *h* in Fig. 2a), *ϕ* and $\tilde{\phi }$ are linked by the relationship, $\phi ={h}^{-1}\circ \tilde{\phi }\circ H$. As mentioned before, since epithelial sheets are two-dimensional structures, what we directly estimate here from positional cell data is the 2D map $\tilde{\phi }$, and its 3D representation *ϕ* is ultimately reconstructed using that relationship (see Fig. 3 for the flowchart of tissue deformation analysis). In practical estimation processes, a map is modeled by a lattice deformation; i.e., the correspondence of positional coordinates before and after deformation for each lattice point are estimated (see the *gray* lattice in Fig. 2a as an example, and Supplementary Notes 2, 3). The destination of an off-lattice point is approximated by a weighted sum of the destinations of neighboring lattice points (Supplementary Notes 2, 3).

Once the map is obtained, local tissue deformation can be calculated from the deformation gradient tensor $\tilde{{\boldsymbol{F}}}$ (Fig. 2a and Supplementary Note 2); in particular, quantifying the spatial patterns of area growth rate (the change in the area per given time interval) and deformation anisotropy (biased stretching of local tissue pieces) clarifies when, where, and to what extent characteristic deformations occur. On the cellular scale, such local deformation is realized by different processes such as cell proliferation, cell growth, cell death, and cell intercalation. Quantifying local deformation patterns enables us to predict or narrow down the cellular mechanisms that determine organ-specific morphology^2, 19^.

### Bayesian reconstruction of tissue deformation maps

As previously described, total cell recording is often difficult for many vertebrate organs, and discrete snapshot data from randomly and sparsely labeled cells are more practically available. Such positional data of cells include different types of noise, such as stochasticity in cell trajectories originating from the randomness of cell division orientation, and positional rearrangement through push-and-pull dynamics between neighboring cells, as well as measurement noise. Thus, to reconstruct tissue deformation dynamics from such data, statistical approaches are necessary. In this study, we adopted a hierarchical Bayesian modeling, where the statistical parameter ***θ*** to be estimated from the positional data of labeled cells was the discretized tissue deformation map, i.e., the positional coordinate of each lattice point after deformation (Supplementary Fig. 2). Here, we briefly explain the basic concepts and main assumptions in the process of estimating the 2D map $\tilde{\phi }$ (see Supplementary Note 3 for the details of the estimation process).

For a given data point ***u*** (i.e., the 2D positions of labeled cells after deformation), the posterior probability for the parameter has the form *P*(***θ***|***u***) ∝ *P*(***u***|***θ***)*π*_1_(***θ***|***η***_1_)*π*_2_(***η***_1_|***η***_2_). *P*(***θ***|***u***) is the probability of observing the data point ***u*** for a fixed parameter set ***θ***. Conversely, as a function of ***θ*** with ***u*** fixed, this acts as a likelihood function. In regards to this data distribution, we assumed additive noise obeying isotropic Gaussian distribution on the tangent plane at each point in the 3D representation (Fig. 2c and Supplementary Fig. 2). The key point here is that this noise distribution needs to be transformed into its 2D representation for modeling *P*(***u***|***θ***), and the transformed distribution is no longer isotropic but anisotropic due to the anisotropy of the metric $\tilde{g}$ as shown earlier (Fig. 2a, b). For each data point (*i*-th point), the variance-covariance matrix ${\tilde{\Sigma }}_{(i)}$ and the metric have the following relationship:

2 $${\tilde{\Sigma }}_{(i)}={\sigma }^{2}{\tilde{g}}^{-1}(\tilde{\phi }({{\boldsymbol{U}}}_{i}))\cong {\sigma }^{2}{\tilde{g}}^{-1}({{\boldsymbol{u}}}_{i}),$$

Where *σ* ^2^ is the magnitude of the noise. In the computation, ${\tilde{\Sigma }}_{(i)}$ was approximated by the metric at the data point after deformation (i.e., at ***u***_*i*_) as shown in the rightmost term in Eq. (2) because $\tilde{\phi }$ itself is the value to be estimated and thus it is unknown before computation. As will be described later, including this noise anisotropy into the data distribution model is important for improving the estimation performance. *π*_1_(***θ***|***η***_1_) and *π*_2_(***η***_1_|***η***_2_) are the prior distributions for the parameter ***θ*** and for the hyper-parameter ***η***_1_. ***η***_2_ is also the hyper-parameter necessary for determining the shape of the distribution *π*_2_. As biologically plausible prior information, we assumed the smoothness of deformation both inside the tissue as well as at its boundary. Specifically, the first spatial derivative of the deformation gradient tensor $\tilde{{\boldsymbol{F}}}$ and the second derivative of the boundary curve are assumed to be not large (Supplementary Figs. 2 and 3, and see also Supplementary Note 3 for the concrete implementation).

Using these conditions, in the computation the values of the hyper-parameters are first determined by maximizing the marginal likelihood function ∫*P*(***u***|***θ***)*π*(***θ***|***η***)d***θ***; then, using these values, the discretized deformation map ***θ*** is obtained by maximizing the posterior probability^13, 20, 21^. Finally, from the relationship $\phi ={h}^{-1}\circ \tilde{\phi }\circ H$, the 3D deformation map is reconstructed.

### Validation of the proposed method using artificial data sets

**Fig. 4:**
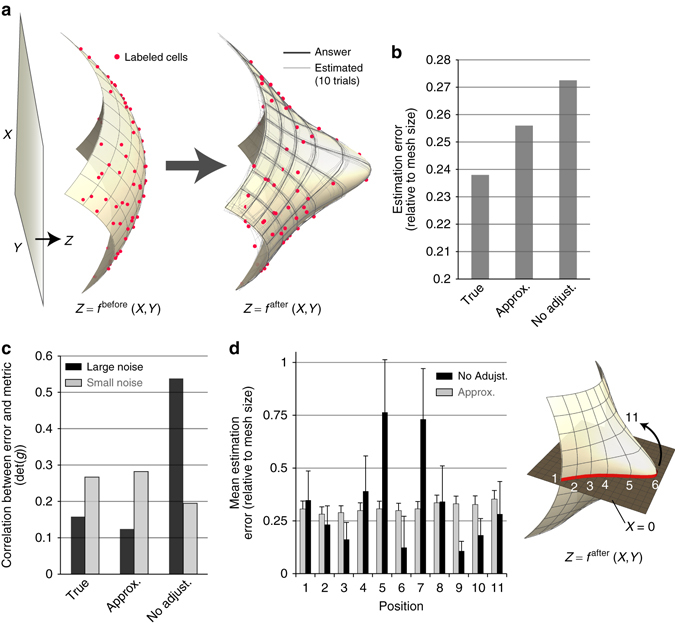
Validation using artificially generated data I. **a** A test of how the estimation performance is affected by spatially heterogeneous noise anisotropy resulting from metric anisotropy. We Inferred the deformation map of a surface whose shape before and after deformation can be represented as graphs. **b** Since the answer of the map was given, the performance of the proposed method was evaluated by its estimation error (see Supplementary Note 4–1). The labels, “True”, “Approx.”, and “No adjust.” indicate cases in which the noise anisotropy was calculated using the answer of the map given a priori (Eq. (2)), was approximated using the data point (Eq. (2)), or was not taken into consideration (i.e., 2D isotropic noise was assumed), respectively. Including the noise anisotropy in the likelihood improved the mean estimation error by 5–10%, but more importantly, **c** it prevented spatially biased error; otherwise the error strongly depends on position (more precisely, the error is highly correlated with the size of the induced metric that is measured by $\det [\tilde{g}]$), which was clearly observed when the magnitude of noise was not negligible. **d** Positional dependence of the mean estimation error over 10 estimation trials. The error bar indicates the standard deviation. Incorporating metric-dependent noise anisotropy into the statistical model improves the error bias. In particular, the error in the region with a steep gradient (e.g., at position 5) was clearly improved

**Fig. 5:**
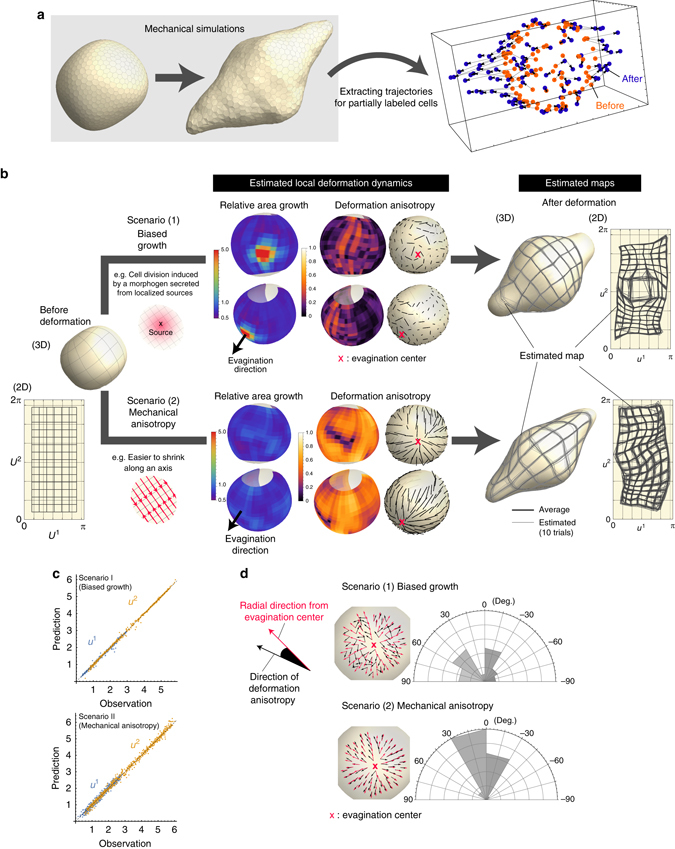
Validation using artificially generated data II. **a** A test of how the estimation performance is affected by spatially heterogeneous noise anisotropy resulting from metric anisotropy. Test data were generated by mechanical simulations of elastic membranes (see Supplementary Note 4–2 for the details of calculation). **b** Two potential candidates for tissue evagination were simulated: biased tissue growth (*upper*) and mechanical anisotropy without growth (*lower*). Local deformation dynamics (specifically, the area growth and deformation anisotropy) for both mechanisms were precisely reconstructed and could be clearly distinguished. In the biased growth model, the large area expansion in the growth region was correctly detected, while the degree of deformation anisotropy was low over the whole domain and showed no significant trend in its direction. In the mechanical anisotropy model, spatial heterogeneity of the area growth rate could barely be detected, and deformation anisotropy along the evagination axis was clearly observed throughout the entire tissue. **c** The performance of the proposed method was evaluated by examining the prediction errors (cross validation) as well as residual errors. The small prediction error assured the validity of the proposed method. The *blue* and *yellow dots* indicate results for the *u*_1_ and *u*_2_ coordinates, respectively. **d** In addition to the spatial pattern of the area growth rate, the distribution of the direction of deformation anisotropy around the evagination center can also be a clear signature for distinguishing both mechanisms of tissue evagination

We evaluated the performance of our method by using artificially generated data (Figs. 4 and 5). Considering the later biological application to forebrain morphogenesis in which OV formation is one of the main events, we generated test data from two types of tissue evagination models.

The first model depicts the evagination of a surface whose shape before and after deformation can be represented as graphs, i.e., *z* = *f*(*x*, *y*) (Fig. 4a and see also Supplementary Note 4-1 for details). In this case, the 2D coordinates for each cell within the sheet can be simply given by its projection onto the *z*-plane. The deformation map is defined by specifying the correspondence of positions before and after deformation for all points on the sheet. The data generated from this model were used to test how estimation performance is affected by the spatial heterogeneity of noise anisotropy resulting from the heterogeneity of the metric tensor whose values are determined by the choice of the 2D coordinate system (Fig. 2). To analyze this, we compared the following two situations: one case in which isotropic noise was assumed in the data distribution *P*(***u***|***θ***), and another case in which noise anisotropy was adjusted based on the metric tensor at each position (Eq. (2)). As shown in Fig. 4b, the average estimation error was only improved by 5–10% by including the noise anisotropy, but more importantly, we found that when the noise anisotropy is not included in the likelihood function, the estimation precision of deformation dynamics depends on the position within the tissue; for some domains, their deformation dynamics is more precisely estimated, but in other domains it is worse (Fig. 4c, d). This position-dependent estimation error was evident when the magnitude of noise added to the test data was not negligible (reflecting real biological data), while it was barely detectable when the noise was relatively small (corresponding to a subcellular scale). This analysis clearly showed that modeling the data distribution with metric-dependent noise anisotropy is necessary for preventing bias in the estimation error, or in other words, for preventing the dependence of choosing the 2D coordinate system on the sheet. In regard to the magnitude of the estimation error itself, compared to the mesh size, deformation maps and spatial patterns of deformation characteristics are captured precisely.

Next, in order to generate data reflecting more realistic situations, we performed mechanical simulations for evagination of an elastic membrane (Fig. 5 and see also Supplementary Note 4-2 for details). In the simulations, two potential candidate mechanisms for achieving tissue evagination were tested: biased tissue growth as seen in plant root development^22^, and mechanical anisotropy without growth, such as the main mechanism in Drosophila germband extension^23, 24^ (Fig. 5b). The main purpose was to evaluate whether these two different deformation mechanisms can be clearly distinguished using only positional data from sparsely labeled cells. Different from the previous case, only the rules for growth rate or mechanical anisotropy were given, and for each cell the position after deformation was not specified a priori, meaning that the value for the deformation map *ϕ* was not given. Therefore, the performance of the proposed method was validated indirectly by examining prediction errors (i.e., cross validation).

In the inference processes, the 2D coordinates for the cells were given using the SHE, and taking into consideration the morphological symmetry, the deformation map for the region that included one protrusion was estimated. As shown in Fig. 5b,c, deformation dynamics for both mechanisms were precisely reconstructed and could be clearly distinguished from each other. In particular, the average prediction errors were relatively small compared to the mesh size representing the deformation map, thus demonstrating the high performance of our method (Fig. 5c). In the model of mechanical anisotropy, spatial heterogeneity of the area growth rate was barely detected, and deformation anisotropy along the evagination axis was clearly observed throughout the entire tissue. Specifically around the evagination center, radial tissue elongation was observed (Fig. 5d). In contrast, in the biased growth model, the large area expansion in the growth region was detected correctly, while there was no significant trend (nearly random) in the direction of deformation anisotropy (Fig. 5d). These results demonstrated that the directional distribution of the deformation anisotropy, especially around the evagination center, as well as that of the area growth rate, can be a clear signature for distinguishing different mechanisms of tissue evagination.

Furthermore, we also found that the number of data points comparable to the number of lattice points used to approximate the deformation map can provide satisfactory estimation performance. This provides useful information for application to actual biological data, as the fineness of the mesh and the number of data points necessary can be estimated to achieve the desired precision. Additionally, the analysis described above demonstrates that our continuum approach to the inference process works well even if the morphological change includes slight discontinuity due to positional rearrangement between the discrete points that correspond to the cell centers.

### Application to chick forebrain morphogenesis

Many vertebrate organs are formed from epithelial tissues. From the simple morphology that initially exists, region-specific tissue deformation leads to the generation of organ- and species-specific morphology. Early forebrain morphogenesis is a good model for studying how complex morphology is generated from a simple sheet-like structure (Fig. 1). During this process, the most dramatic morphological change is the formation of the OV. After its formation, an eye (i.e., optic cup and lens) will be formed at the tip of OV. Since local tissue deformation is realized by different cellular processes, early histological studies of OV formation focused on cellular characteristics such as height and diameter using fixed mouse and chick samples. Specifically, it was reported that cell height decreases during the evagination process^25, 26^. However, how such cellular morphological changes contribute to tissue-level deformation or determine overall forebrain shape was difficult to discuss.

In more recent works, taking advantage of the small size and transparency of embryos, four-dimensional imaging of the evagination process in the teleost OV was performed^27–31^. In the study by Ivanovitch *et al*.^32^, characteristic cellular behavior during evagination of the zebrafish OV was shown. Prior to OV evagination, two types of cells exist in the forebrain: the marginal cells which form a cylindrical layer structure and have an apicobasal polarity like an epithelial cell, and the core eye field cells that are found inside the tube formed by the marginal cells and which have a mesenchyme-like shape. It was observed that the mesenchyme-like core cells intercalated into the layer of marginal cells during OV evagination through a mesenchymal-epithelial transition. However, it remains unknown whether this intercalation itself is a driving force of tissue evagination/elongation or not. To understand if and how such local cellular behavior contributes to morphogenesis, it is essential to quantify tissue-level deformation dynamics and clarify the relationship between cellular and tissue-level events, the latter of which, as described above, is often lacking.

### In vivo validation of the deformation analysis

Here, we applied the methods for tissue deformation analysis that we proposed above to data from chick forebrain morphogenesis. We chose to study chick embryos since, in contrast to teleost embryos, their forebrain morphogenesis can be regarded as the deformation of a simple single-layered sheet. Using two-photon microscopic imaging, we measured the 3D morphology of the entire forebrain and the positional changes of sparsely labeled cells at every somite stage. Cell-labeling was done by adding glutathione-coated quantum rods (Q-rods)^33, 34^ that can randomly attach to apical surfaces. More precisely, we focused only on small aggregations of Q-rods ranging from 1 to 20 micrometers in diameter, and the positions of such Q-rod aggregates were manually tracked (see Methods and Supplementary Fig. 8 for details of the experiments). The deformation of both dorsal and ventral surfaces was analyzed from SS5 to SS10, and from SS10 to SS13 only the dorsal surface was analyzed due to limitations in imaging resolution.

**Fig. 6:**
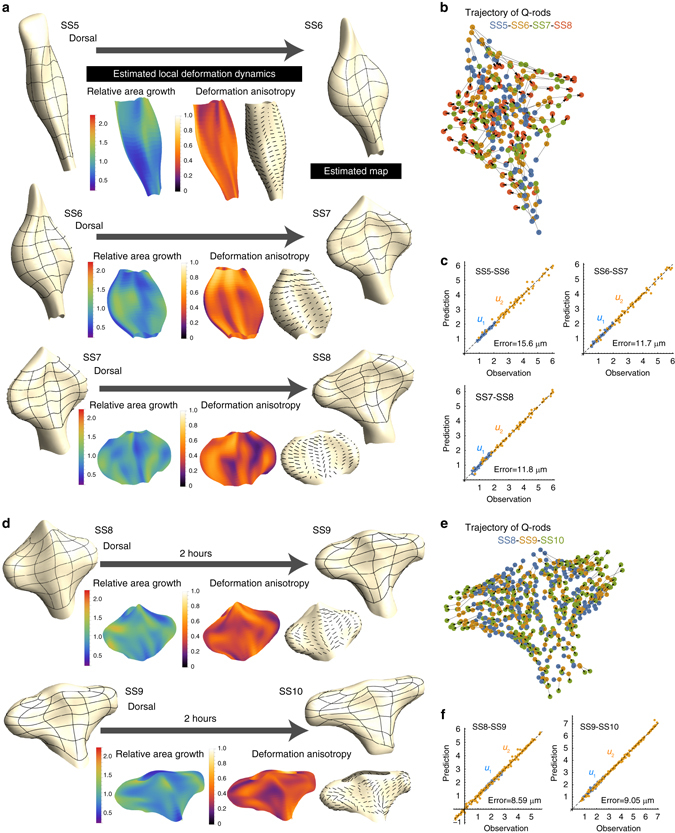
Application to chick forebrain morphogenesis from SS5 to SS10. **a**, **d** Estimated deformation maps and spatial patterns of local deformation characteristics from SS5 to SS8 for one embryo **a** or from SS8 to SS10 for a different embryo **d**; the figures show deformation of the dorsal apical surface (see Supplementary Figs. 9–11 for results on the deformation of the ventral apical surface, and the results for other embryos). No clear bias in the area growth rate was observed even with slight variations among positions. However, the deformation anisotropy was uniformly high throughout the entire forebrain and its direction was aligned along the medio-lateral axis (shown as *black line* segments on the surface). These tendencies were consistent from SS5 to SS10 (and still held until SS13, shown in Fig. 7). **b**, **e** Trajectory data from small aggregates of Q-rods (ranging 1–20 micrometers in diameter) attached to the apical surface that were used to infer the tissue deformation map. **c**, **f** The precision of the estimated maps was evaluated by prediction errors. The mean prediction error was on the order of a few cell diameters (on the apical surface). The *yellow* and *blue dots* indicate results for the *u*_1_ and *u*_2_ coordinates, respectively

**Fig. 7:**
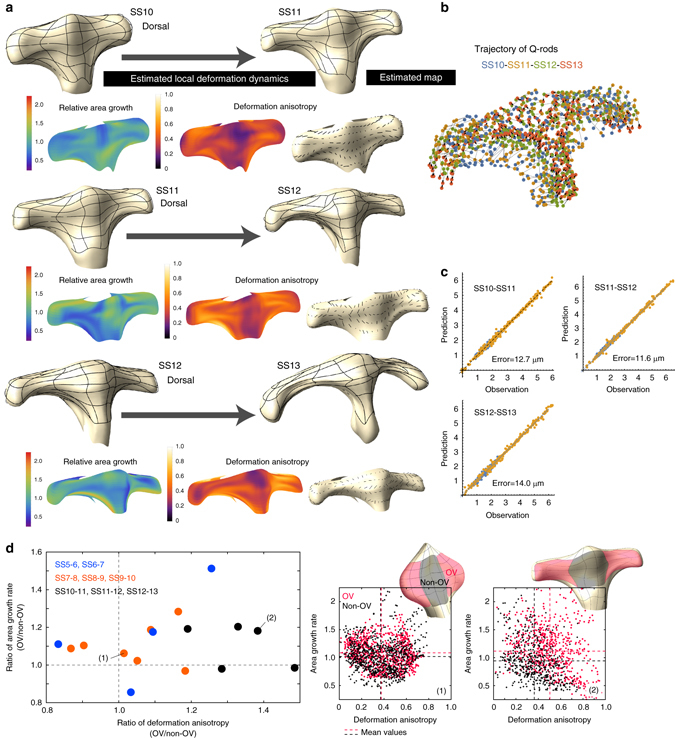
Application to chick forebrain morphogenesis from SS10 to SS13. **a** Estimated deformation maps and spatial patterns of local deformation characteristics from SS10 to SS13 for one embryo. Due to limitations in the imaging resolution, deformation dynamics were only analyzed for the dorsal apical surface (see Supplementary Fig. 11(D) for results from another embryo). As with the case for SS5-SS10, no clear bias in area growth rate was observed. In regards to deformation anisotropy, its value remained high especially in the OV region (or evaginated region). During these somite stages, differences in the degree of anisotropy were clearer between OV and non-OV regions (quantified in (**d**)). The direction of deformation anisotropy was generally biased along the medio-lateral axis although the pattern was growing more complex with the increasing morphological complexity. **b** Trajectory data from small aggregates of Q-rods (ranging 1–20 micrometers in diameter) attached to the apical surface that were used to infer the tissue deformation map. **c** The precision of the estimated maps was evaluated by prediction errors. The mean prediction error was on the order of a few cell diameters (on the apical surface). The yellow and blue dots indicate results for the *u*_1_ and *u*_2_ coordinates, respectively. **d** Quantitative comparison of tissue deformation characteristics on the dorsal surface between OV and non-OV regions. The *left* graph shows the ratio of the average degree of deformation anisotropy and the ratio of average area growth rate between these two regions. Each point shows a result for each reconstructed deformation map. The difference in deformation anisotropy between OV and non-OV regions was clearer at later stages. The *right panels* show examples of distributions of deformation anisotropy and area growth rate at different positions for two maps ((1) and (2) in the *left panel*)

As with the second example of in silico validation described in the previous section, the 2D coordinates on the neuroepithelial sheet were given by the SHE. Figures 6 and 7 show the results of the deformation analysis (see also Supplementary Figs. 9–11). We first checked the estimation performance; the prediction error was around 10 μm, which correspond in size to the diameter of a few cells and were small enough compared to the size of the mesh used to estimate the tissue deformation map (Figs. 6c, f and 7c). This clearly shows the applicability of the proposed method to actual experimental data.

### Tissue deformation patterns and cellular characteristics

**Fig. 8:**
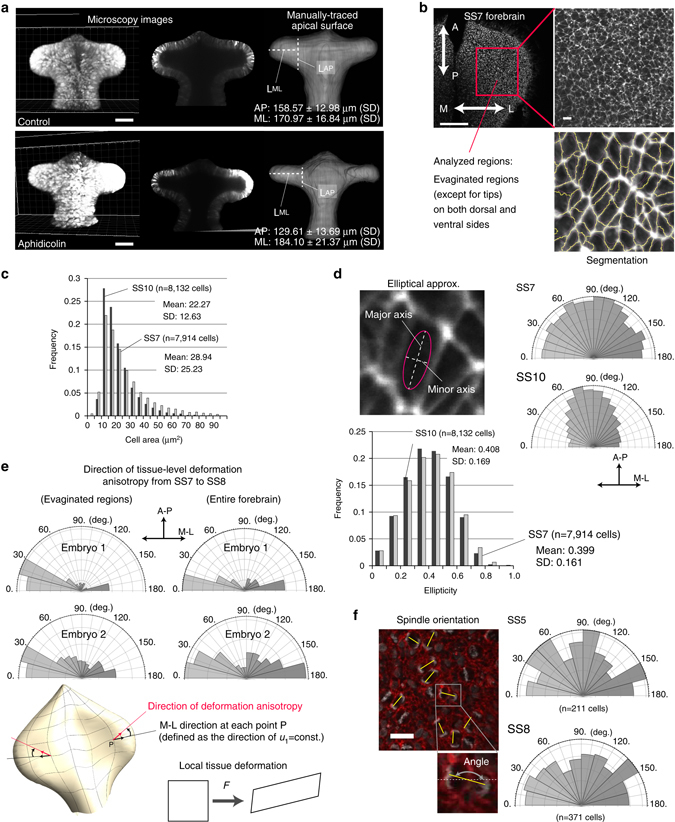
Growth inhibition and quantification of cellular characteristics. **a** Evagination of the optic vesicle still occurred when cell proliferation was inhibited by aphidicolin (see also Supplementary Fig. 13). Scale bar: 100 μm. **b** Regions analyzed for the quantification of cellular characteristics (specifically, cell area **c**, cell shape **d**, and division orientation **f**). Scale bars: 100 μm (*upper left* in **b**), 10 μm (*upper right* in **b**). **c** Mean cell size differed slightly depending on stage and was smaller during later stages, but the variance was large. Since the area of the evaginated region gradually increased, it was concluded that changes in cell size did not contribute to morphogenesis. **d** Cell shape was basically elliptical (far from a rounded shape) and its degree was almost constant at different stages (SS7 and SS10). In contrast, the direction of the major axis of cell shape changed over time; during earlier stages of evagination it was almost randomly distributed, while it had a bias in the anteroposterior direction. However, as shown in **e**, the direction of anisotropy of local tissue deformation had a clear bias along the medio-lateral axis. Consequently, the orientation of the major cell shape axis could not explain the anisotropic tissue deformation. **f** The direction of division orientation was almost random, and also failed to explain the anisotropic tissue deformation. Taken together, the quantification of cellular characteristics suggests that forebrain morphogenesis, especially optic vesicle formation, is driven by cellular rearrangement. Scale bar: 20 μm

We then examined the spatio-temporal patterns of the deformation characteristics. As for the area growth rate of the apical surface, no clear bias was observed throughout the entire period we analyzed (SS5-SS13), although some variations were present depending on position. In contrast, deformation anisotropy showed a clear pattern, with high values and globally oriented direction along the medio-lateral axis throughout the entire period of forebrain analysis although, at later stages (e.g., after SS11), the definition of the M-L axis became ambiguous due to the morphological complexity and the differences in the degrees of deformation anisotropy for the OV and non-OV regions became clearer. These results clearly show that anisotropic deformation (i.e., biased tissue stretching) along the medio-lateral axis, and not local area growth, is the predominant mechanism underlying the dynamic change in forebrain morphology during the period of our focus, which we think could not be readily predicted by simply comparing the morphologies shown in Fig. 1d. This was also supported by the experiment in which cell proliferation was inhibited using aphidicolin (Fig. 8a and Supplementary Fig. 13). Although total size was slightly reduced, evagination itself was still clearly observed, demonstrating that cell proliferation contributes to the size of the OV but is not important for the evagination process.

Furthermore, we examined the changes in tissue thickness during morphogenesis. We first confirmed that, consistent with previous histological studies, tissue thickness and cell height itself gradually decreased over time (from 65 μm at SS5 to 52 μm at SS13, on average). At each stage, the distribution of thickness was narrow (Supplementary Fig. 7). We then calculated the rate of thickness change per somite stage at each position (Supplementary Fig. 12), and found that there was almost no correlation with the area growth rate of the apical surface throughout the entire study period. From these, we concluded that changes in tissue thickness or cell height do not contribute to morphogenesis during early development of the forebrain.

At the cellular level, we quantified apical area, shape, and division orientation within the evaginated regions (Fig. 8 and Methods). While the mean apical cell area was slightly smaller at later stages, its variance was quite large compared to the mean and we did not find any specific patterns. The major axis of cell shape was almost randomly distributed at earlier phases (e.g., SS7), whereas after the OV became clearer (SS10), direction was biased in the anteroposterior axis, perpendicular to the direction of OV evagination (i.e., the medio-lateral axis). Within the corresponding region, tissue-level deformation anisotropy trended remarkably in the direction of the medio-lateral axis (Fig. 8e), demonstrating that deformation anisotropy could not be explained by changes in cell shape. Similar to the major cell axes, tissue anisotropic deformation could not be explained by division orientation, as its distribution was also random (Fig. 8f). Based on this quantification of cellular characteristics, changes in cell size and shape or division orientation were excluded as potential candidate determinants of anisotropic tissue deformation. Our experiments and analyses thus suggest that the dynamic morphological changes in the forebrain are driven by positional rearrangement of the cells. Results from the mechanical simulations shown above (Fig. 5) also suggest that mechanical anisotropy is present (i.e., such as the anisotropy in stress distribution and/or in mechanical tissue properties) and drives cellular rearrangement along an axis. Experiments on mechanical perturbation and observations of its response will be important and interesting future issues for identifying the driving forces that achieve the tissue deformation dynamics described in this study.

## Discussion

Here, we have demonstrated a differential-geometrical and Bayesian method for reconstructing tissue deformation dynamics for 3D morphogenesis of curved epithelial sheets from positional data of landmarks. Unlike previous studies in which velocimetric analysis using high-resolution data was performed, our approach enables precise reconstruction from data with limited spatiotemporal resolution, expanding the possibility of tissue-level deformation analysis for many vertebrate organs for which total cell recording is difficult. Furthermore, our method would also be useful for analyses during later stages of development. Compared to early development, little is known about tissue deformation dynamics during later development. A major reason for this limitation is that organ size often becomes larger than 1 mm in scale for which high-resolution live imaging of developing tissues is quite difficult using confocal and two-photon microscopy. In contrast, although the spatial resolution is much lower (0.1–1 mm) than in microscopes, a larger range of tissues (1–100 mm) can be scanned using magnetic resonance imaging, computed tomography, or ultrasound imaging^35^. In these cases, the method proposed here could contribute to the analysis of such low resolution data.

Our investigation has also shown that the quantitative analysis of tissue deformation dynamics is useful for predicting mechanisms that determine organ-specific morphology. As demonstrated in the example of tissue evagination, spatial patterns of deformation characteristics, such as directional distribution of deformation anisotropy and surface expansion, show clear signatures for distinguishing background mechanisms that generate similar morphologies. In particular, during chick forebrain development, strong and globally oriented deformation anisotropy along the medio-lateral axis was observed throughout the entire region as well as relatively non-biased area growth patterns. This strongly suggests that the evagination is driven mainly by cell intercalation along a specific axis, not by local growth, which was confirmed by additional experiments involving the inhibition of cell proliferation and quantification of cellular characteristics.

The anisotropic deformation through dynamic cellular rearrangement implies the existence of anisotropic stress distribution within the tissue. Previous studies on epithelial tissue development of different organs/species have identified some factors for generating mechanical anisotropy, these include the localization of phosphorylated Myosin^23, 24, 36, 37^ and PCP proteins^38–40^, and mechanical constraints such as external forces and boundary conditions^41–44^. Quantitatively linking such force generation mechanisms with tissue deformation dynamics would enable the development of models for explaining morphological anomalies and diversity among species, and would aid the morphological design of artificially constructed organoids.

Furthermore, during the analysis of major cell shape axis orientation, we found that in the evaginated region, the axes were perpendicular to the direction of anisotropic tissue deformation (Fig. 8d). This tendency is commonly seen in other systems where cell rearrangement induces directional tissue elongation. Xenopus gastrulation is one such example^45^. Another example is Drosophila germband extension, evident in mutants in which posterior midgut invagination does not occur (specifically, the tor mutant)^4, 43^. In that case, without posterior midgut invagination, the extrinsic or boundary force is diminished, while cell rearrangement and tissue elongation still occur, although to a lesser degree than in wild types. Does this tendency toward a directional relationship between cell shape and cell rearrangement share a common mechanism among different systems? This would also be an interesting issue to study from a mechanical viewpoint.

As previously described, the teleost OV is composed of two types of cells, epithelial-like marginal cells and mesenchyme-like core cells, and during OV evagination the core cells vertically intercalate into the layer of marginal cells, which is considered to contribute to OV formation. In contrast, in amniotes such as chick, the eye field is composed of a single layer of neuroepithelial cells. Our current analysis suggested that the intercalation of epithelial cells within this layer is the predominant cellular mechanism that induces anisotropic tissue deformation. Despite differences in the initial cell arrangements and the direction of cell intercalation between chick and zebrafish, both embryonic tissues elongate along a common medio-lateral axis. Comparative analysis of the molecular mechanisms that induce the evagination process in both embryos will be an interesting future topic from an evolutionary perspective.

## Methods

### Embryos

Fertilized chicken eggs (Shiroyama Farm) were incubated in a humidified incubator at 38 °C to obtain Hamburger and Hamilton (HH) stage 4 embryos^46^.

### Construction of fluorescent protein expressing plasmid

To label the rostral portion of the neural tube, we used fluorescent protein expressing plasmids containing the N2 enhancer of Sox2^47^. We amplified the myrVenus gene (fluorescent protein with myristoylation signal peptide myr) by PCR, using following primers: myrVenus forward 5′-AAGCTTATGGGAAGCAGCAAGAGCAAGCCAAAGATGGTGAGCAAGGGCGAGG-3′ and myrVenus reverse 5′-TCTAGA TTACTTGTACAGCTCGTCC-3′. Using these fragments, we replaced the *EGFP* gene in the pN2-EGFP^47^ with the myrVenus gene resulting in pN2-myrVenus.

### Culture preparation and electroporation

The embryos were explanted using the EC culture method^48^ with some modifications. For electroporation and 3D imaging, the vitelline membrane was removed in 123 mM NaCl solution. For neural epithelial cell labeling, we electroporated pN2-myrVenus plasmids into the future neural tube region. We used a CUY21EX electroporator (BEX) at the following settings: poration pulse 7 V, 1 pulse of 50 ms. Afterward, 3 V driving pulses, five pulses of 50 ms with a 50 ms interval.

### Labeling of apical surface using quantum rod

For labeling the apical surface, we used two approaches. In one method, we positioned the electroporated embryo with the dorsal side up, and added a drop of Glutathione-coated Q-rods^33^, then incubated the embryo in a CO_2_ incubator (37 °C, 5% CO_2_) until the desired stage. The addition of a drop of glutathione-coated Q-rods was done prior to neural tube closure. At that stage, the apical surfaces of the neuroepithelial cells are exposed on the outside of the embryo, thus we could label the apical, not basal, surface. In the other method, we first incubated the electroporated embryo in a CO_2_ incubator (37 °C, 5% CO_2_) until the desired stage. Then, we introduced Q-rods inside the neural tube using a glass capillary tube (tissue was pierced from a posterior approach so it did not affect the morphogenesis of the forebrain region). Note that, at target stages, the neural tube was already closed. The latter method was more efficient for labeling tissues, that is more Q-rods (precisely, more small aggregations of Q-rods) were attached to the apical surfaces. In particular, the latter method was necessary for introducing sufficient numbers of small Q-rod aggregations at later stages (after SS10).

The size of each Q-rod used was <100 nanometers, but objects chosen for analysis in our study only included small aggregations of Q-rods with a diameter of 1–20 micrometers (corresponding to a scale from the subcellular level to the diameter of a few cells) that were clearly detected by our microscopic set-up and distinguishable from one another in the manual tracking process. The small aggregations tended to remain on the apical surface.

### Multi-photon live imaging and data processing

Live 3D imaging was performed using an upright microscope (FV1000MPE; Olympus) equipped with an Olympus 25×/NA1.05 XLPLN25XWMP objective and a multi-photon femtosecond laser (excitation wave length 920 nm; Mai-Tai DeepSeeeHP, Spectra-Physics). For the live imaging, an electroporated embryo was immersed in 1× PBS. The 3D morphology of the neural tube was determined by stacking the optical section images along the Z-axis (at an interval of 5 μm with a total of 80 sections). For each Z-level, four X-Y images (512 × 512 pixels each) were tiled so that the entire region of the prospective brain was included. The embryo was then returned to culture until the next imaging time point. All images were taken at room temperature. Imaging intervals (incubation time) were 2 h. Within each interval, embryo development progressed to the next somite stage. Although this developmental speed is slightly slower than that in normal development (1.5 h/somite), the morphology itself appeared normal.

Initial image processing was done with Imaris 7.6 (Bitplane). In order to obtain the 3D morphology, we first manually traced the apical and basal surfaces of the neural tube for each section along different axes. By stacking the traced data, the 3D morphology of the rostral neural tube lumen was reconstructed. The result of manual tracing could be obtained as a set of dots, as shown in Fig. 1b (middle), from which the 3D models of the apical and basal surfaces and 2D coordinates on the apical surface were obtained using the SHE (see Supplementary Fig. 6 for the validation of the approximation of both apical and basal surfaces by SHE). Using the 3D models, the tissue thickness was measured (Supplementary Fig. 7). The position of the Q-rods (small aggregations ranging from 1 to 20 micrometers in diameter) attached to the apical surface before and after deformation were manually linked (Supplementary Fig. 8), and their 2D coordinates were obtained by projecting them onto the approximated surface by the SHE.

### Deformation analysis

From the positional data of the Q rods, the 2D deformation map was estimated using the proposed method. The analytical procedure was similar to that used for the validation with artificially generated data (see Supplementary Note 4-2-4). The deformation analysis was performed for six different embryos in total.

### Inhibition of cell proliferation

In Fig. 8a, for the inhibitor treatment, embryos were grown in an EC culture setting (dorsal side up) until somite stage (SS) 6 then treated with aphidicolin (Sigma-Aldrich) dissolved in PBS to a final concentration of 100 μM. Embryos were then returned to culture until the desired stage. See Supplementary Fig. 13 for the confirmation that cell cycle progression was efficiently inhibited by treatment with aphidicolin.

### Quantifying forebrain morphology and cell characteristics

In Fig. 8a, for the quantification of forebrain morphology, embryos were grown until somite stage (SS) 10, and the forebrain was imaged using a multi-photon microscope. The cavity size of the OV along the medio-lateral and the anterior-posterior axes was measured using Fiji software after projecting the 3D image in the z-plane. In Fig. 8b–d, for the quantification of apical cell shape and the angle of cell division in the OV regions, embryos were fixed for 1 h with 4% PFA in PBS at room temperature. The neural tubes were dissected into dorsal and ventral halves and permeabilized with 0.5% Triton X-100 (NacalaiTesque) in PBS for 30 min at room temperature. F-actin was stained with phalloidin, using a standard protocol. The neural tubes were mounted with their apical sides up in Fluoroshield with 4',6-diamidino-2-phenylindole (DAPI) (ImmunoBioScience). Images of the stained actin fiber networks were acquired with a FV1000 confocal microscope (Olympus). A standard watershed algorithm in Fiji software was used to identify cell boundaries. From the segmented image of each cell, its apical area was quantified. Based on the variance in the position of cell boundary pixels, cell shape (ellipticity) was calculated as follows:

$${\rm{Ellipticity}}=1-{\lambda }_{\min }/{\lambda }_{\max },$$

Where *λ*_max_ and *λ*_min_ are the larger and smaller eigenvalues, respectively. The direction of cell shape was evaluated by that of its major axis. Lastly, late-stage mitotic cells (from anaphase to cytokinesis) were identified by DAPI staining. The cell division orientation was quantified as the angle between the medio-lateral axis (along which evagination occurs) and the line through the centers of the two daughter cells. In all Figures of 8a–d and f, statistics were calculated using at least three embryos.

### Data availability

The data that support the finding of this study are available from the corresponding author upon reasonable request.

## Electronic supplementary material

Supplementary Information Supplementary Figures, Supplementary Notes and Supplementary References
