# Supplementary material for: Reconstructing 3D deformation dynamics for curved epithelial sheet morphogenesis from positional data of sparsely-labeled cells
Source: Nat Commun. 2017 May 2;8:15. doi: 10.1038/s41467-017-00023-7 (PMC5432036; doi:10.1038/s41467-017-00023-7)
Supplement: Supplementary file 1 — Supplementary Figures, Supplementary Notes and Supplementary References [file 41467_2017_23_MOESM1_ESM.pdf]

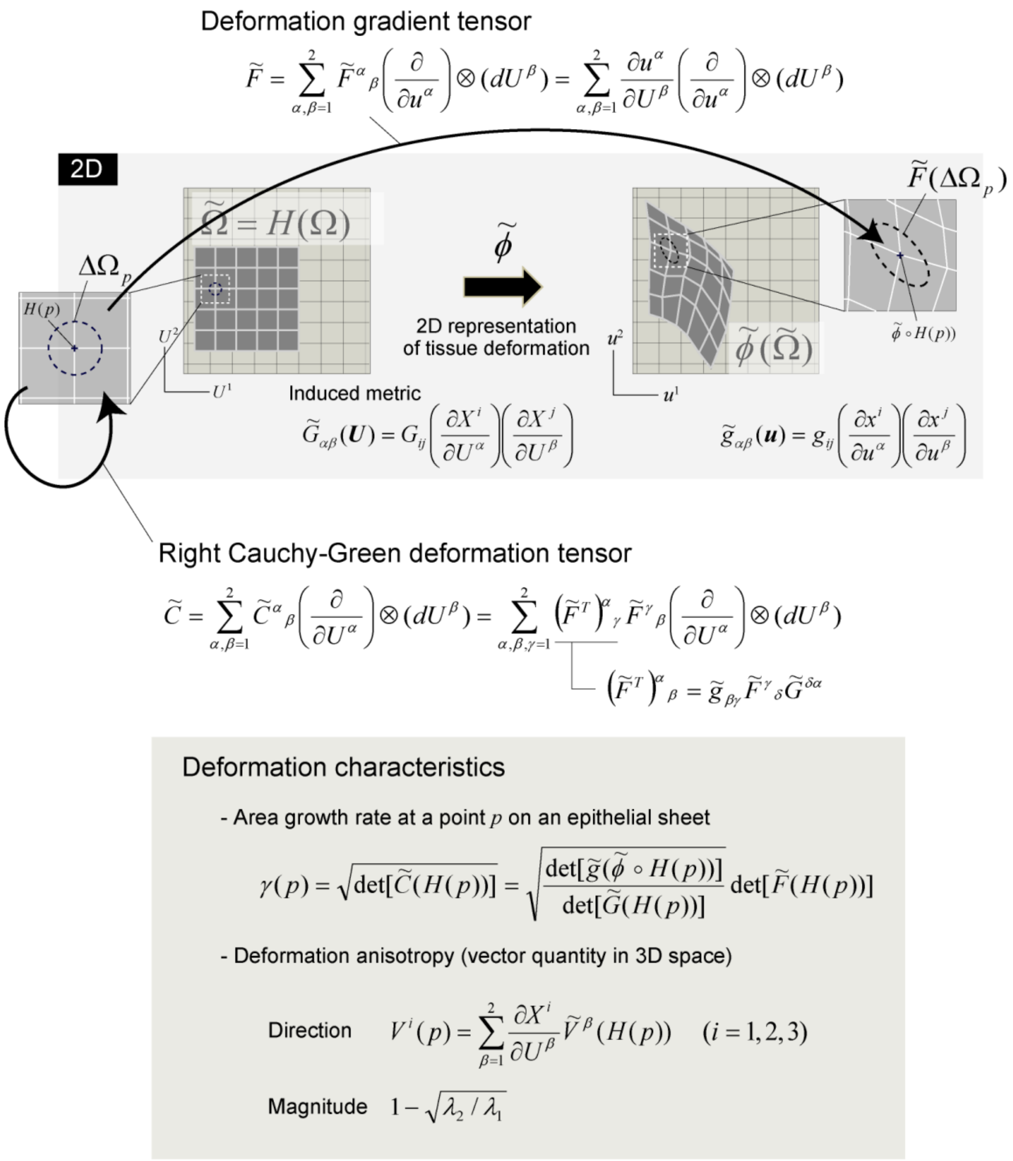

Supplementary Figure 1: The definition of deformation characteristics (see Supplementary Note 2 for details).

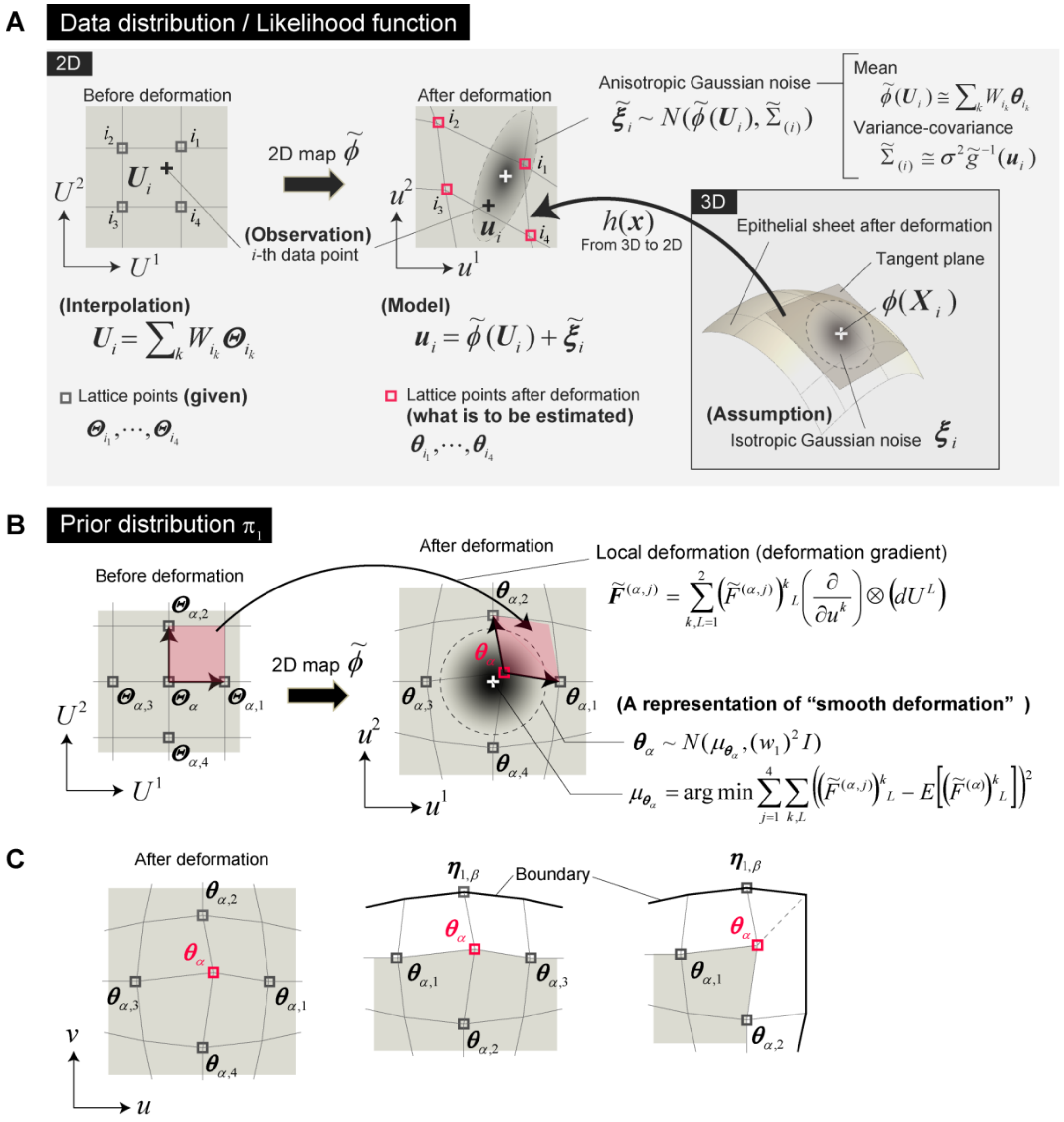

Supplementary Figure 2: Bayesian inference of tissue deformation maps from positional data of landmarks. (A) Likelihood function. In the method proposed in this paper, using positional data of landmarks, a 2D tissue deformation map $\tilde{\phi}$ modeled by lattice deformation is estimated; that is, the 2D coordinate of each lattice point after deformation ($\boldsymbol{\theta}$) is estimated. The observed landmark position after deformation is assumed to include additive noise obeying isotropic Gaussian distribution (ξ_i) on the tangent plane at each point $\phi(\boldsymbol{X}_i)$ in the 3D representation, where ϕ is the 3D representation of $\tilde{\phi}$ and what we ultimately would like to determine. $\boldsymbol{X}_i$ is the positional coordinate before deformation of the i-th landmark. In the 2D representation, the noise distribution becomes anisotropic due to the metric anisotropy ($\tilde{\xi}_i$). That is, in a given 2D coordinate system, the position of i-th landmark after deformation ($\boldsymbol{u}_i$) is modeled as $\boldsymbol{u}_i = \tilde{\phi}(\boldsymbol{U}_i) + \tilde{\xi}_i$, where $\boldsymbol{U}_i$ is the 2D representation of $\boldsymbol{X}_i$. (B) Prior distribution of parameters. The smoothness of the deformation inside the tissue is assumed, which is represented such that the first spatial derivative of the deformation gradient tensor is not large. See Supplementary Note 3-3 for definitions of variables and mathematical details. (C) Neighborhood relationship in the calculation for prior distribution of the parameter $\boldsymbol{\theta}_\alpha$. When the focal lattice point is inside the calculation domain (in gray) for which the deformation map is estimated, the neighboring four lattice points inside the domain are used. In contrast, when the focal lattice point is on the edge of the domain, a boundary point is involved. In particular, if it is at the corner of the domain, only three neighboring points are considered and thus only the two parallelograms spanned by the pairs ($\boldsymbol{\theta}_{\alpha,1} - \boldsymbol{\theta}_\alpha$, $\boldsymbol{\theta}_{\alpha,2} - \boldsymbol{\theta}_\alpha$) and ($\boldsymbol{\theta}_{\alpha,1} - \boldsymbol{\theta}_\alpha$, $\boldsymbol{\eta}_{1,\beta} - \boldsymbol{\theta}_\alpha$) are used for calculating μ_{θ_α} (see Eq. (S23)).

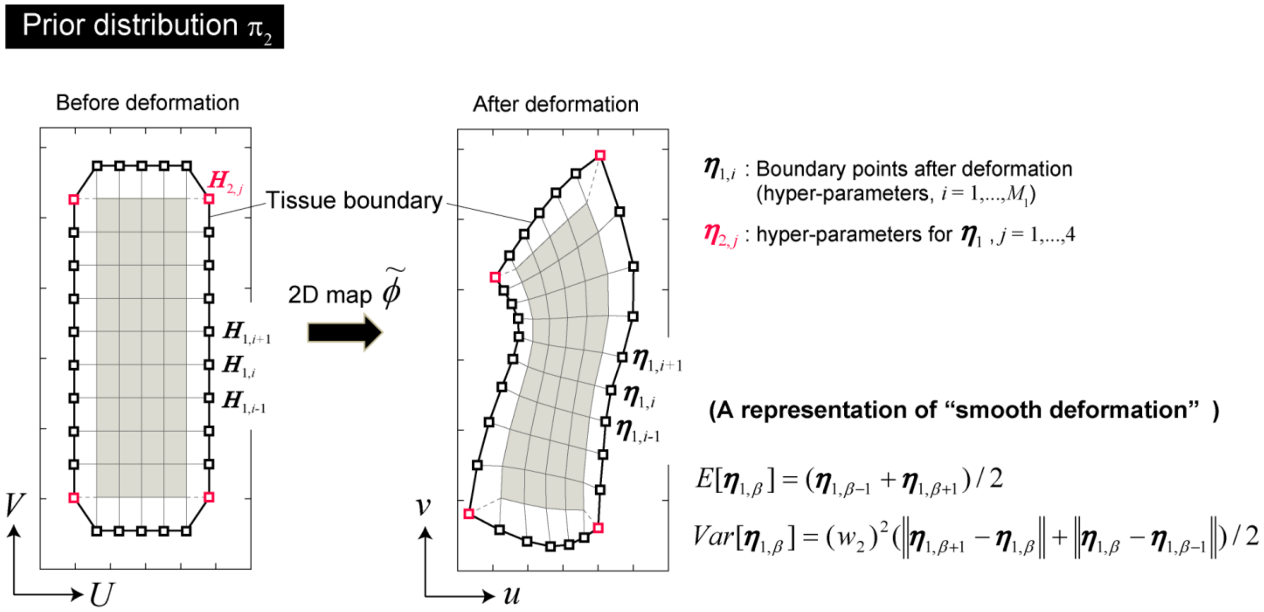

Supplementary Figure 3: Schemes for the prior distribution π_2 (see Supplementary Note 3-3 for the definitions of variables and mathematical details).

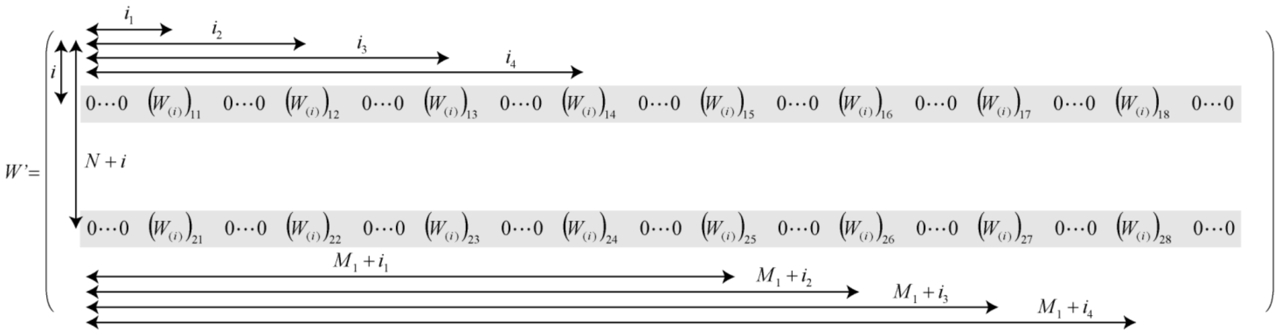

Supplementary Figure 4: The definition of matrix W' in Eq. (S21).

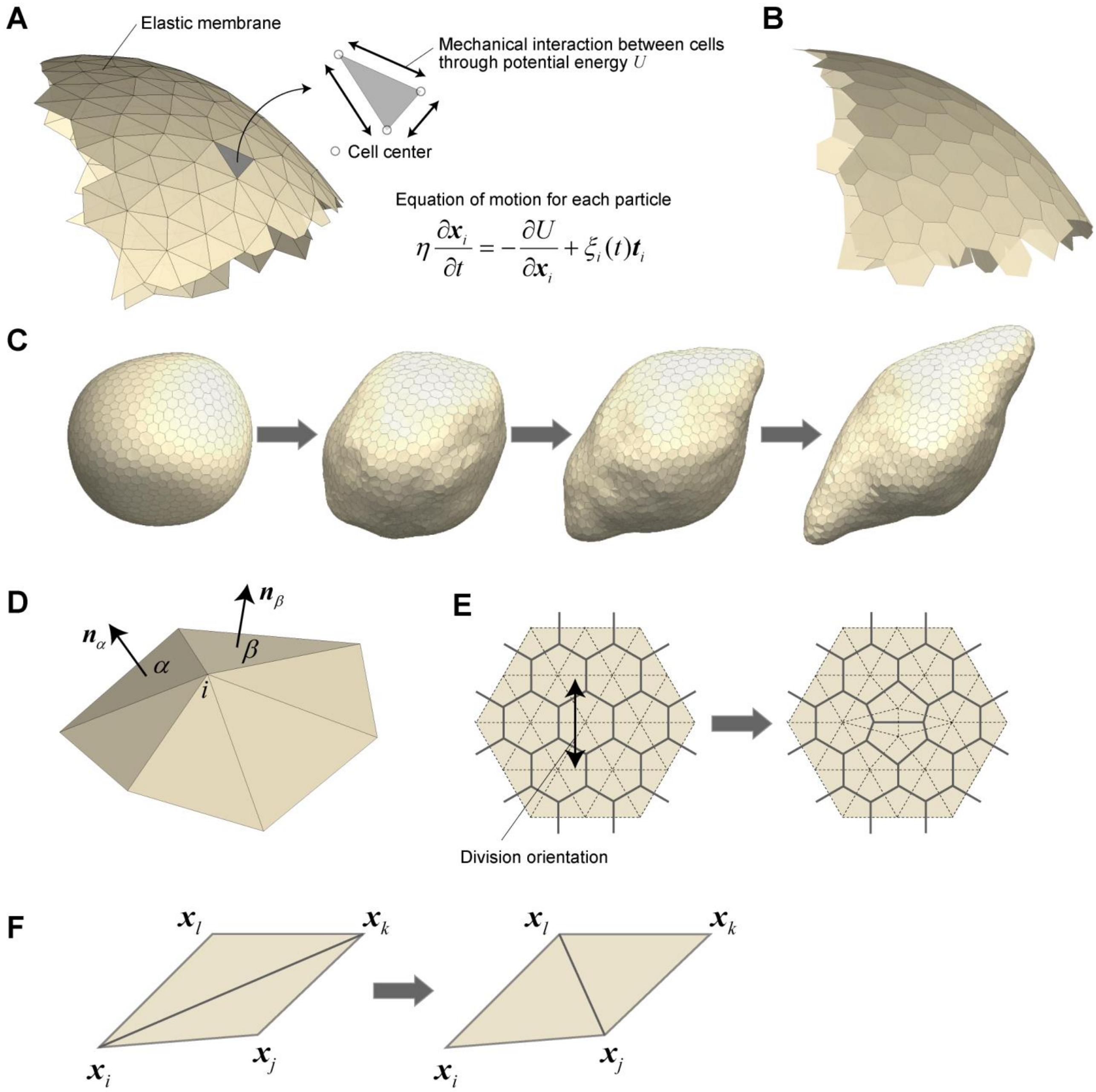

Supplementary Figure 5: Mechanical simulation of elastic membranes in Fig. 5. (A) Each cell is modeled as a particle represented by its center position. Through intercellular potential energy, adjacent cells mechanically interact with each other. (B) Voronoi representation of tissue surface. (C) An example of temporal change in membrane morphology based on scenario II (the mechanical anisotropy model). (D) Normal vectors of each triangular element (see Eq. (S37)). (E) Implementation of cell division. (F) Implementation of the re-mesh process. See also Supplementary Note 4-2.

Validation of approximation of apical and basal surfaces by spherical harmonics expansion

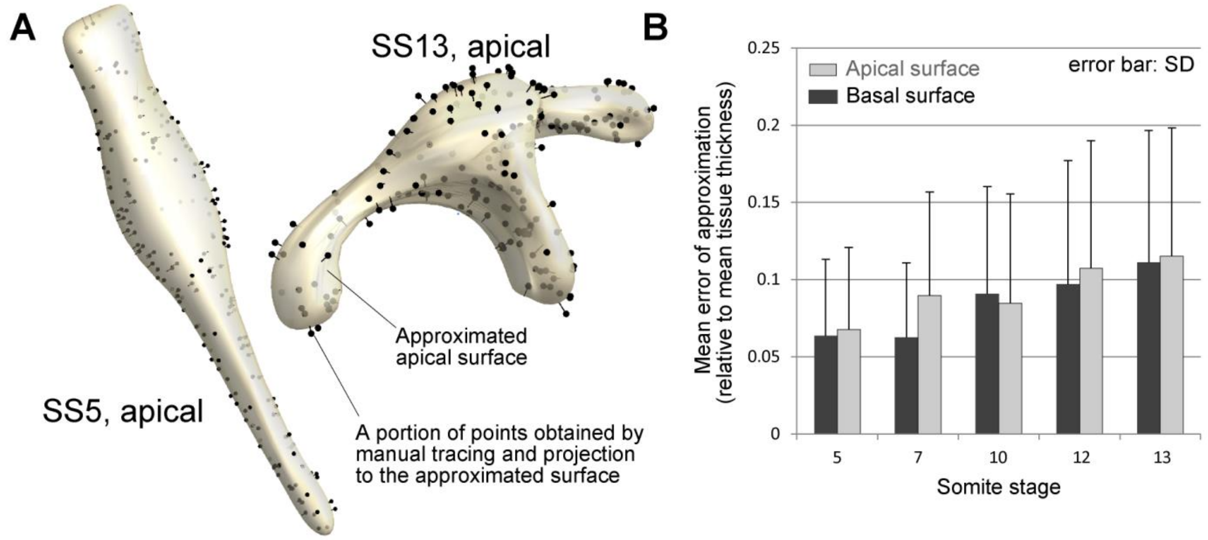

Supplementary Figure 6: Validation of approximation of apical and basal surfaces by spherical harmonics expansion. (A) The validity of the approximation is graphically shown for two different morphologies of apical surfaces at SS5 and SS13. The black points are a subset of points obtained by manual tracing, and the small black segments indicate the projections to the approximated surfaces. (B) The graph shows the quantification of approximation errors. The errors for both the apical and basal surfaces were around 10% of tissue thickness for the entire period of analysis.

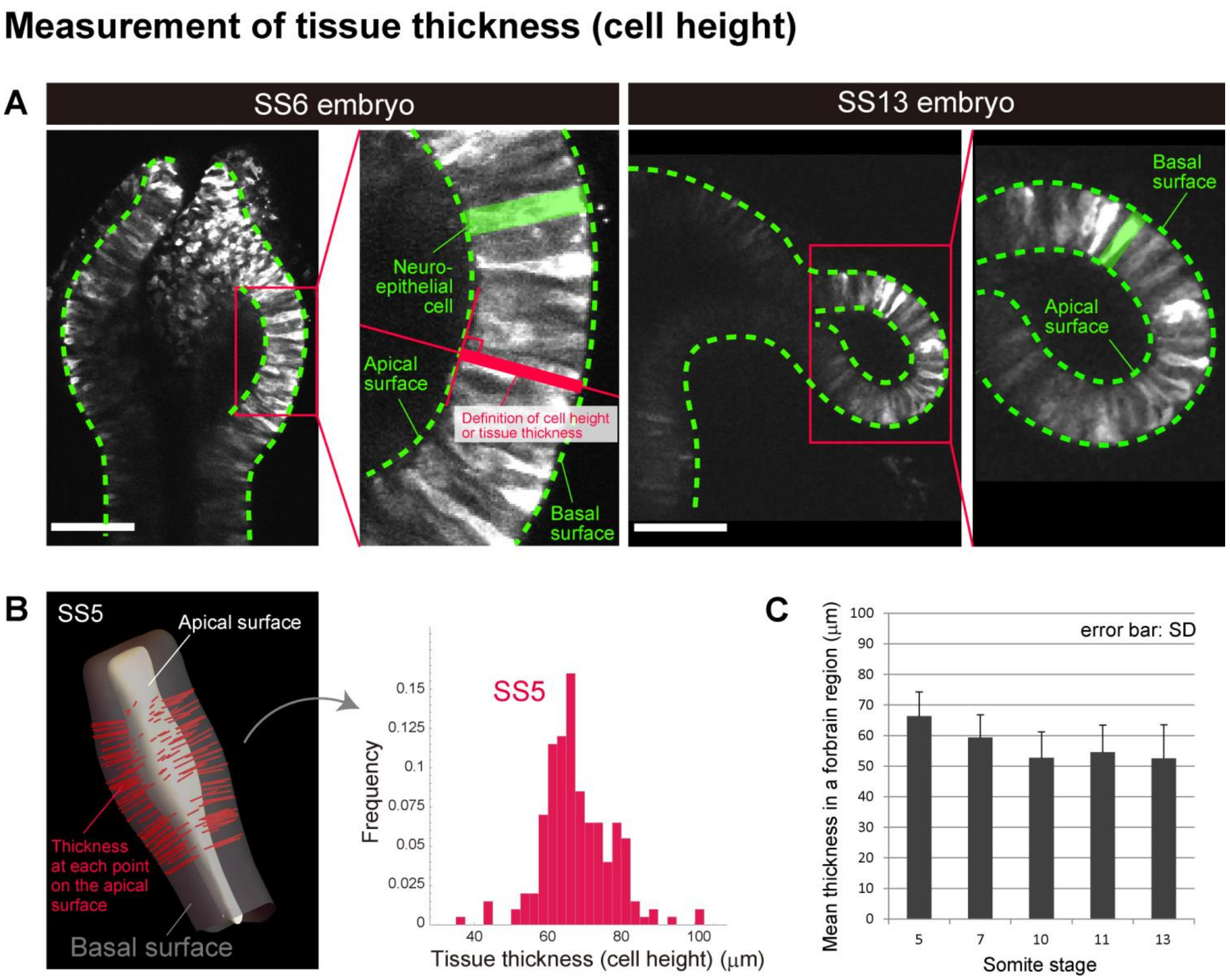

Supplementary Figure 7: Measurement of tissue thickness. (A) Two examples of a portion of the forebrain region at different stages (SS6 and SS13) are shown. The imaging resolution was sufficient to manually trace the apical and basal surfaces by changing the plane of focus in the directions of the different axes. The result of the manual tracing could be seen as a set of dots, as shown in Fig. 1B (middle), from which the 3D models of the apical and basal surfaces were constructed by the spherical harmonics expansion (please see also Supplementary Fig. 6 for the validity of this approximation). Since the neuroepithelial sheet is a monolayer structure, at least during the period we focused on, and cell shape is columnar and its apicobasal axis is almost perpendicular to both surfaces, we could determine tissue thickness or cell height at each position on the sheet by measuring the distance between each point on the apical surface and its projected point on the basal surface. (B) An example of how to measure the distribution of tissue thickness (an example at SS5 is shown). (C) Quantification of mean thickness in the forebrain at different somite stages. At each stage, the distribution was narrow (see the error bar for standard deviation), and its mean gradually decreased with time. Scale bar: 100μm.

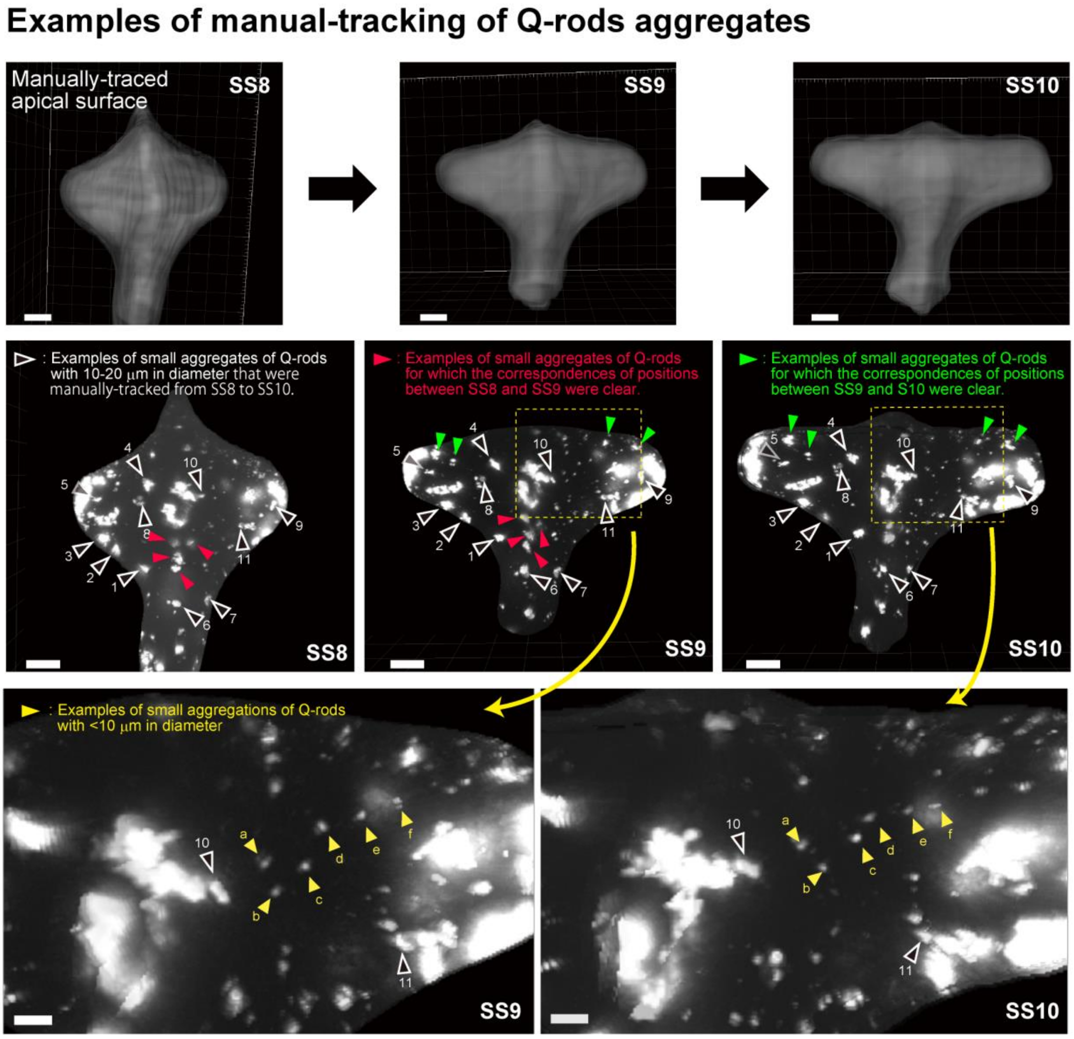

Supplementary Figure 8: Tracing Q-rod aggregates. As landmarks for inferring the tissue deformation maps, we focused on the positional data from small aggregates of Glutathione-coated Q-rods (1-20 micrometers in diameter; about 80% of such aggregates could be clearly distinguished). The diameter of each Q-rod particle is less than 100 nm, which was difficult to detect using the settings of our imaging system. The figure shows an example of manually-traced aggregations of Q-rods. Scale bar: 50μm (top and middle panels), 20μm (bottom panels).

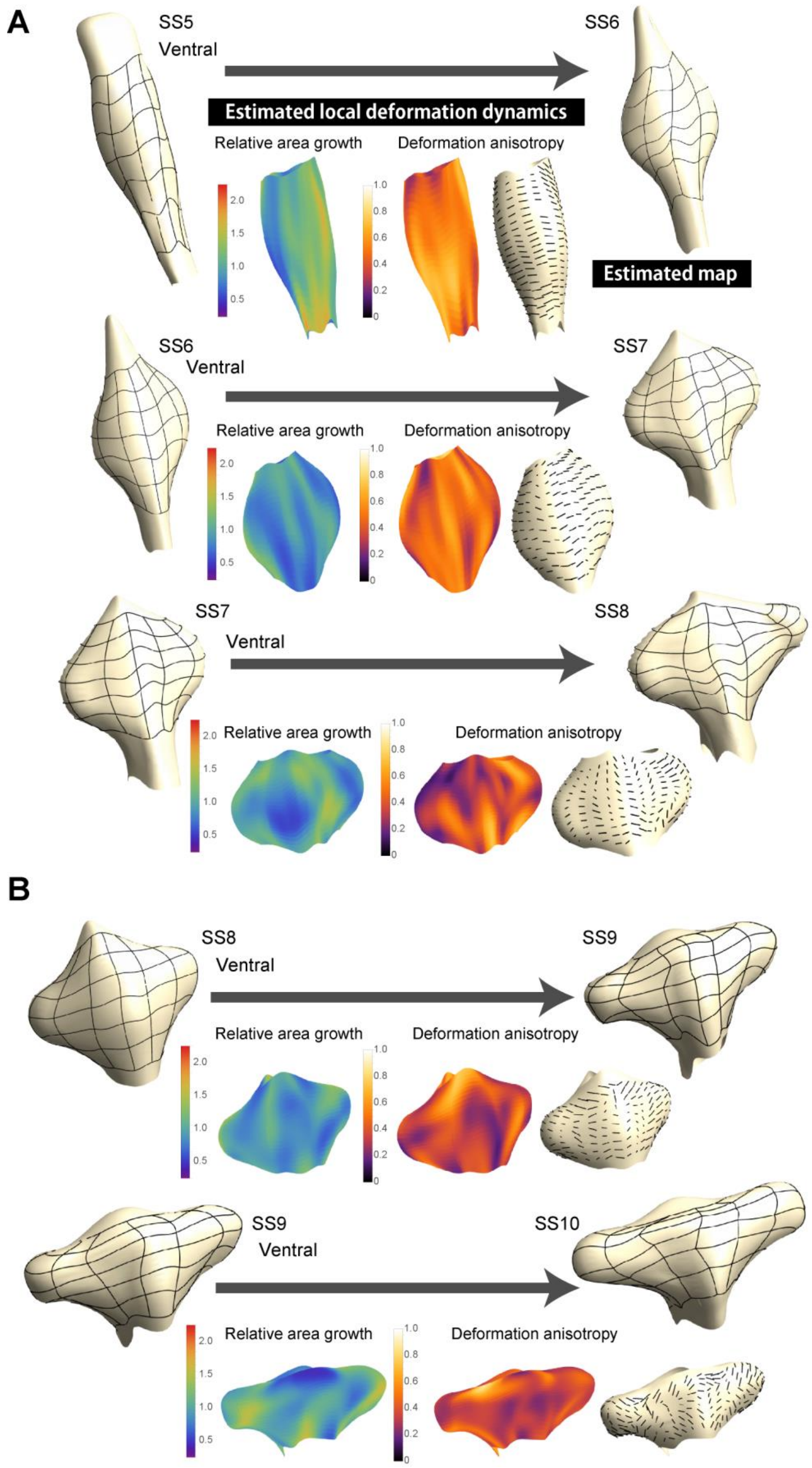

Supplementary Figure 9: Results of tissue deformation analysis on the ventral surface for the two embryos shown in Fig. 6. (A, B) Estimated deformation maps and spatial patterns of local deformation characteristics.

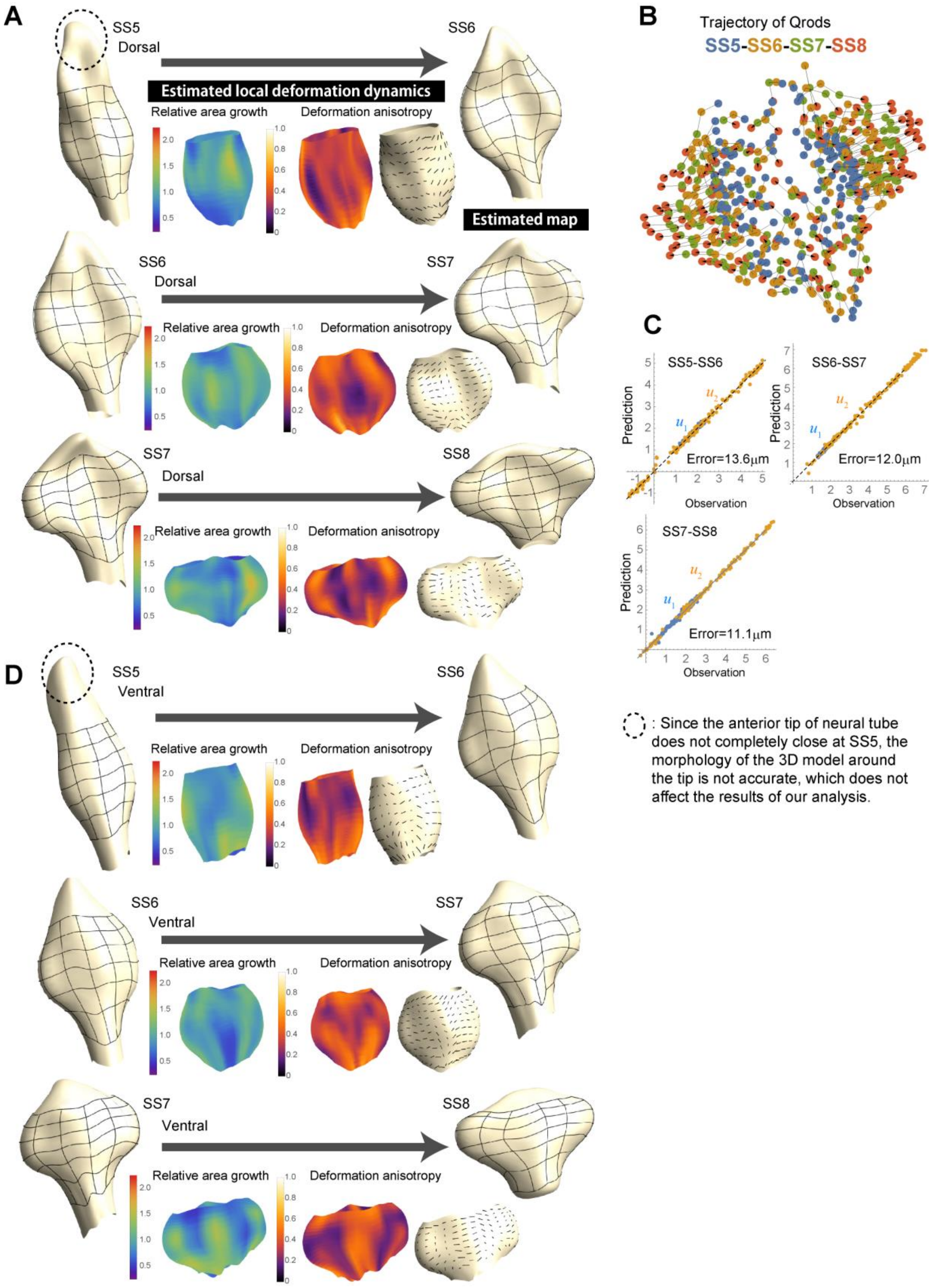

Supplementary Figures 10: Results of tissue deformation analysis for another embryo different from the embryos shown in Figs 6, 7, and Supplementary Figure 9. (A, D) Estimated deformation maps and spatial patterns of local deformation characteristics on the dorsal and ventral surfaces, respectively. (B) Trajectory data of small aggregations of Q-rods attached to the apical surface that were used for inferring the tissue deformation maps. (C) Evaluation of prediction errors.

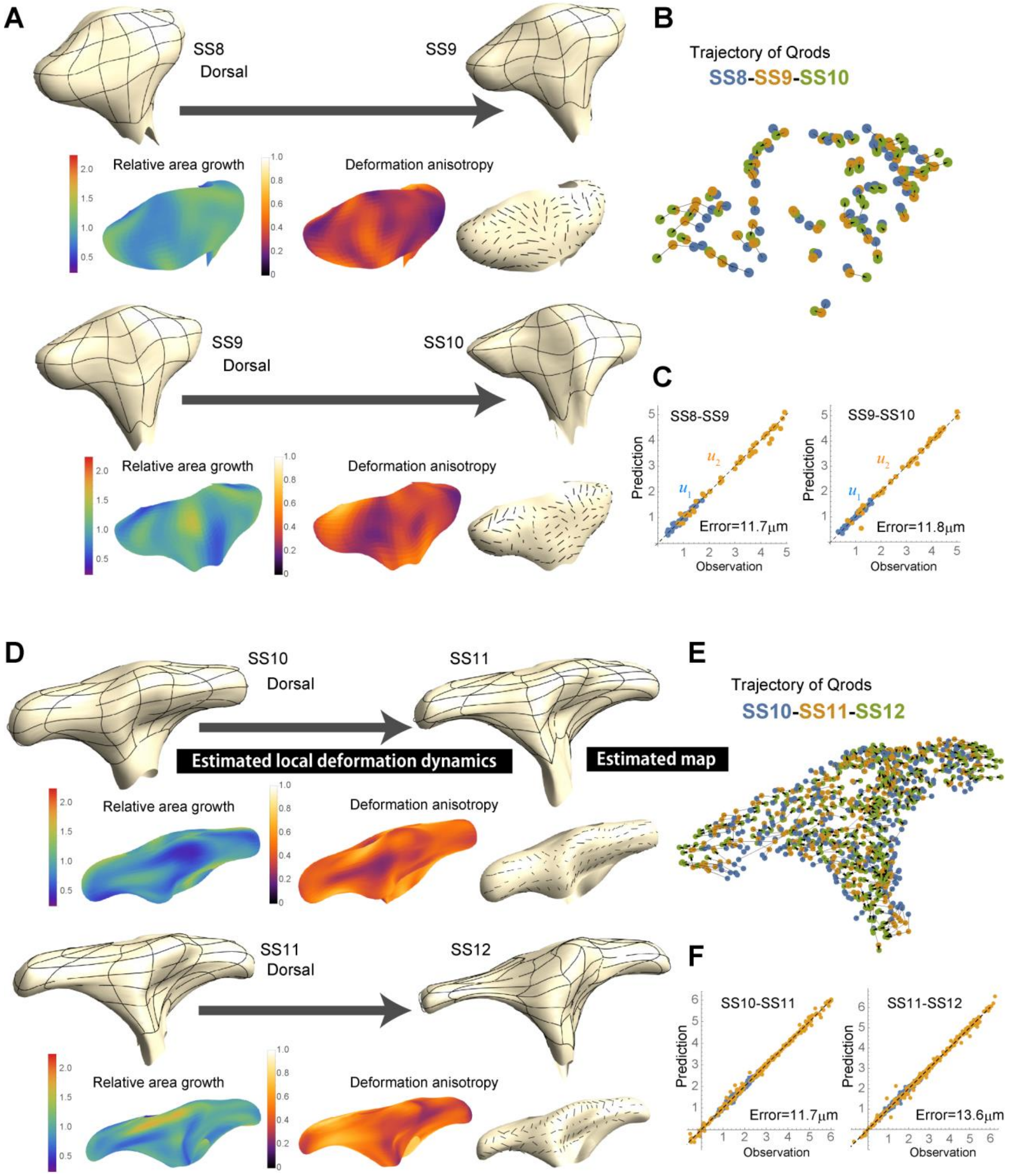

Supplementary Figure 11: Results of tissue deformation analysis for other embryos. (A, D) Estimated deformation maps and spatial patterns of local deformation characteristics on the dorsal surface for two different embryos. (B, E) Trajectory data of small aggregations of Q-rods attached to the apical surface that were used for inferring the tissue deformation maps. (C, F) Evaluation of prediction errors.

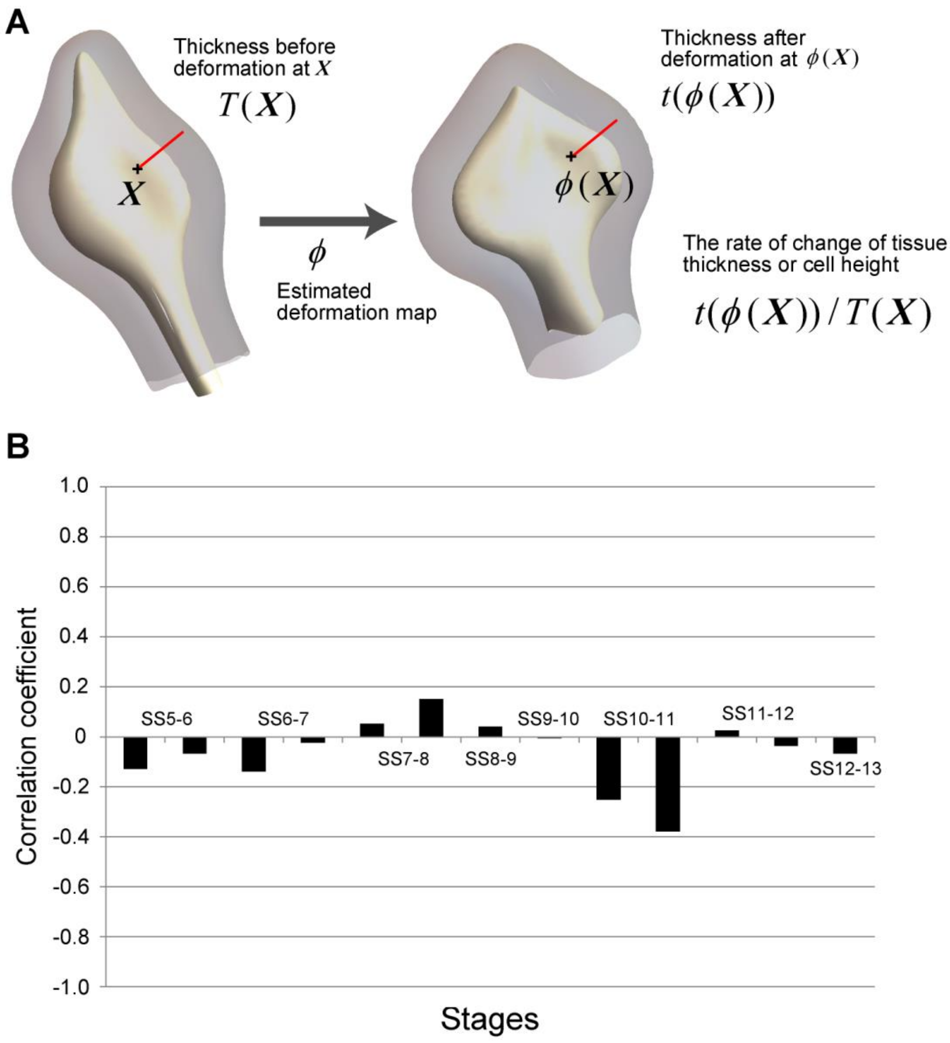

Supplementary Figure 12: Calculation of the rate of change of tissue thickness or cell height. (A) Methods for calculating the rate of thickness change. In the calculation, we used the reconstructed tissue deformation maps to link landmark positions on the sheet between different time points. (B) Correlation coefficients between the rate of thickness change and the area growth rate of the apical surface for different samples at different stages. In all cases, the correlation was low, showing that the change in thickness or cell height does not contribute to morphogenesis during the early development of the forebrain.

Cell cycle progression was efficiently inhibited by the treatment with aphidicolin.

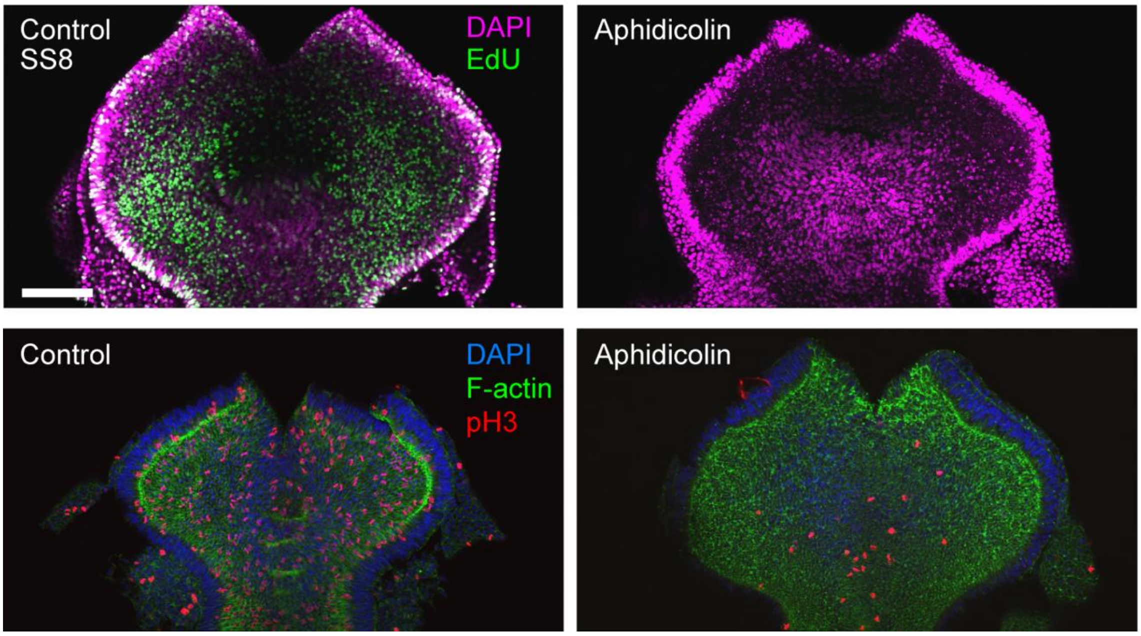

Supplementary Figure 13: Cell cycle progression was efficiently inhibited by treatment with aphidicolin (see Fig. 8A and Methods). Scale bar: 100µm.

Supplementary Note 1: Defining 2D coordinate charts on epithelial sheets

1-1. Coordinate systems on 2D manifolds and examples

As stated in the text, epithelial tissues can often be dealt with as 2D sheets. In mathematical terms, those tissues are regarded as 2D manifolds, thus using appropriate local coordinate systems (e.g., H or h in Fig. 2A), the 2D coordinates specifying each point on the surface of a tissue before deformation $\boldsymbol{U} = (U^1, U^2)$ and after deformation $\boldsymbol{u} = (u^1, u^2)$ can be defined. The corresponding 3D coordinates are given by the inverse of those charts:

$$\boldsymbol{X} = (X^1, X^2, X^3) = H^{-1}(\boldsymbol{U}) \text{ and } \boldsymbol{x} = (x^1, x^2, x^3) = h^{-1}(\boldsymbol{u}).$$

According to standard practice[1,2], quantities before (or after) deformation are denoted with uppercases (or lowercases), and the components of the coordinates are represented as a superscript, for example, (X^1, X^2, X^3) instead of (X, Y, Z), which is a convenient method for representing the summation of the products with components of other vectors and matrices, e.g., $\sum_i A_i X^i$. One of the few exceptions to this rule is the superscript of the variance σ^2 which represents the exponent, and will be clear from the context.

One of the easiest ways of allotting 2D coordinates (i.e., the simplest chart) is with a simple projection, e.g., $\boldsymbol{u} = (u^1, u^2) = (x^1, x^2)$, which is possible when the surface can be described as a single graph, $x^3 = f(x^1, x^2)$. However, as stated in the main text, epithelial tissues often have saclike or tubular morphologies for which representation with a single graph is difficult. Instead, since they can be closely approximated by closed surfaces, the spherical harmonics expansion (SHE) is a more useful way for defining 2D coordinates within them. It should be noted that even if a given surface is not completely closed, such as an open cylinder, the 2D coordinates are still defined on the cylinder by SHE assuming that both ends of the cylinder are closed; coordinates are then also defined on the presumed “caps” at both ends. Using SHE, a closed surface can be approximated by:

$$\boldsymbol{x}(\boldsymbol{u}) = \sum_{l=0}^{L} \sum_{m=-l}^{l} \boldsymbol{\alpha}_l^m Y_l^m(\boldsymbol{u}), \qquad \text{(S1)}$$

where $Y_l^m(\boldsymbol{u})$ is Laplace’s spherical harmonics defined using associated Legendre functions $P_l^m(\cdot)$:

$$Y_l^m(u^1, u^2) = \sqrt{\frac{(2l+1)(l-m)!}{4\pi(l+m)}} P_l^m(\cos u^1) e^{imu^2}. \qquad \text{(S2)}$$

α_l^m is the weighting coefficient for the (l,m)-th harmonics, and is generally a complex number. The approximation becomes more precise by taking higher modes of the harmonics (i.e., using a larger value of L) such as in Fourier series expansion. The software SPHARM is a convenient tool for obtaining the coefficients α_l^m from polygonal mesh data for a given surface[3]. Using the analogy of the relationship between the surface of the earth in 3D space and a 2D world atlas, the component u^1 represents the latitude ranging from 0 to π. While u^2 represents the longitude ranging from 0 to 2π, and the 3D coordinates $\boldsymbol{x}(\boldsymbol{u})$ loop at $u^2 = 2\pi$, i.e., $\boldsymbol{x}(u^1,0) = \boldsymbol{x}(u^1,2\pi)$. Therefore, for points around $\boldsymbol{x}(u^1,0)$ in 3D representation, positional deviations from noise can induce large changes in the value of u^2. In addition, we should be careful that around both poles ($u^1 = 0$ and $u^1 = \pi$ in the 2D representation), small deviations in 3D coordinates can induce large changes in the value of u^2.

When analyzing tissue deformation dynamics, 2D coordinate systems on the surface before and after deformation ($\boldsymbol{U}$ and $\boldsymbol{u}$) can be different and defined independently of each other. How the coordinate systems are chosen is a process completely independent of the tissue deformation dynamics. As will be explained below, the deformation itself defines the relationship between the coordinates on the surface at different time points.

1-2. Induced metric for given 2D coordinate systems

An important point in analyzing surfaces with a large curvature is that the metric in 2D coordinate representation is different depending on the position. As will be explained in the following section, the metric tensor defined at each point plays a key role in calculating deformation characteristics and in modeling data distribution in the estimation process of the deformation map. The component values of the metric tensor depend on the 2D coordinate systems (H and h) that are adopted. Assuming a Euclidean metric (i.e. the Kronecker delta δ_{ij}) in 3D space, the induced metric tensors in the 2D representation (denoted by $\tilde{G}$ before deformation and $\tilde{g}$ after deformation) are given by pull-back operations in regards to maps H^{-1} and h^{-1} as follows:

$$\tilde{G}_{\alpha\beta}(H(p)) \equiv (H^{-1})^{*}\delta_{ij}(p) = \sum_{i,j}\delta_{ij}(p)\left(\frac{\partial X^i}{\partial U^\alpha}\right)\left(\frac{\partial X^j}{\partial U^\beta}\right), \qquad \text{(S3)}$$

$$\tilde{g}_{\alpha\beta}(h\circ\phi(p)) \equiv (h^{-1})^{*}\delta_{ij}(\phi(p)) = \sum_{i,j}\delta_{ij}(\phi(p))\left(\frac{\partial x^i}{\partial u^\alpha}\right)\left(\frac{\partial x^j}{\partial u^\beta}\right), \qquad \text{(S4)}$$

The asterisk symbol (*) is the pull-back operation; p and $\phi(p)$ are 3D positions on the surface before and after deformation, respectively. ϕ is the deformation map in the 3D coordinate system (Fig. 2). The tilde symbol $\tilde{\bullet}$ is used for quantities in the 2D representation.

Supplementary Note 2: Characterization of surface deformation dynamics

Using the above methods, 2D coordinate systems on epithelial tissues before and after deformation ($\boldsymbol{U}$ and $\boldsymbol{u}$) are allotted independently of each other. The tissue deformation map $\tilde{\phi}$ defines the correspondence relationship between both coordinates (Fig. 2A). The main purpose of this paper is to infer $\tilde{\phi}$ from positional data of randomly and sparsely labelled cells, and using the estimated $\tilde{\phi}$, the tissue deformation map for 3D representation, ϕ, is reconstructed from the relationship $\phi = h^{-1} \circ \tilde{\phi} \circ H$ (Fig. 2A). In the following, we explain how to calculate deformation characteristics such as tissue growth rate and deformation anisotropy after obtaining $\tilde{\phi}$.

2-1. Calculation of deformation characteristics for curved surfaces

Once the 2D map $\tilde{\phi}$ is obtained, local tissue deformation can be quantified from the deformation gradient tensor $\tilde{F}$ and the right Cauchy-Green deformation tensor $\tilde{C}$ defined as follows (Supplementary Fig. 1):

$$\tilde{F} = \sum_{\alpha,\beta=1}^{2} \tilde{F}^{\alpha}{}_{\beta} \left(\frac{\partial}{\partial u^{\alpha}} \right) \otimes (dU^{\beta}) = \sum_{\alpha,\beta=1}^{2} \frac{\partial u^{\alpha}}{\partial U^{\beta}} \left(\frac{\partial}{\partial u^{\alpha}} \right) \otimes (dU^{\beta}), \tag{S5}$$

$$\tilde{C} = \sum_{\alpha,\beta=1}^{2} \tilde{C}^{\alpha}{}_{\beta} \left(\frac{\partial}{\partial U^{\alpha}} \right) \otimes (dU^{\beta}) = \sum_{\alpha,\beta,\gamma=1}^{2} \left(\tilde{F}^{T}\right)^{\alpha}{}_{\gamma} \tilde{F}^{\gamma}{}_{\beta} \left(\frac{\partial}{\partial U^{\alpha}} \right) \otimes (dU^{\beta}). \tag{S6}$$

It should be noted that when the metrics $\tilde{G}$ and $\tilde{g}$ are not equal to δ_{ij}, the transpose of $\tilde{F}$ is defined as $\left(\tilde{F}^{T}\right)^{\alpha}{}_{\beta} = \tilde{g}_{\beta\gamma} \tilde{F}^{\gamma}{}_{\delta} \tilde{G}^{\delta\alpha}$ (when both metrics are δ_{ij}, $\left(\tilde{F}^{T}\right)^{\alpha}{}_{\beta} = \tilde{F}^{\beta}{}_{\alpha}$)[2]. $\tilde{F}$ describes the relationship between neighborhoods before and after deformation at each point on the tissue (Fig. 2A and Supplementary Fig. 1); mathematically, it is a linear map between tangent spaces before and after deformation. In contrast, $\tilde{C}$ is a linear map within the tangent space before deformation.

As stated in the main text, the area growth rate (a scalar denoted by γ) and deformation anisotropy (a vector denoted by $\boldsymbol{V}$) are key quantities summarizing the pattern of local tissue deformation. We note that these quantities are on the tissue scale. On the cellular scale, the area growth rate is primarily determined by the balance between cell proliferation, cell growth, and cell death, while deformation anisotropy can be induced by the total effect of direction-dependent cellular behaviors such as cell intercalation and oriented division[4]. For the given 2D coordinate charts H and h, the area growth rate at a point p on the sheet can be calculated as follows:

$$\gamma(p) = \sqrt{\det[\tilde{C}(H(p))]},$$

$$= \sqrt{\frac{\det[\tilde{g}(\tilde{\phi} \circ H(p))]}{\det[\tilde{G}(H(p))]}} \det[\tilde{F}(H(p))]. \qquad \text{(S7)}$$

As for the deformation anisotropy, its direction is given by:

$$V^i(p) = \sum_{\beta=1}^{2} \frac{\partial X^i}{\partial U^\beta} \tilde{V}^\beta (H(p)) \quad (i = 1, 2, 3), \qquad \text{(S8)}$$

where $\tilde{V}^\beta$ is the principal stretch (i.e., the eigenvector associated with the larger eigenvalue) of the deformation tensor $\tilde{C}$, and V^i is the push-forward of $\tilde{V}^\beta$ by map H^{-1} (i.e., $\boldsymbol{V}$ is a vector in 3D space). The magnitude of anisotropy is given by $1 - \sqrt{\lambda_2 / \lambda_1}$ (or $\sqrt{\lambda_1 / \lambda_2}$), where λ_1 and λ_2 ($\lambda_1 \geq \lambda_2 > 0$) are eigenvectors of $\tilde{C}$. As described in the next subsection, when the right Cauchy-Green deformation tensor $\tilde{C}$ (for 2D representations) is defined as the pull-back of its counterpart for 3D representation C, i.e.,

$$\tilde{C}_{\alpha\beta} = \sum_{i,j} C_{ij} \left(\frac{\partial X^i}{\partial U^\alpha} \right) \left(\frac{\partial X^j}{\partial U^\beta} \right) \qquad (i, j = 1, 2, 3), \qquad \text{(S9)}$$

$\boldsymbol{V}$ is the eigenvector of C.

In the special case where a focal sheet is completely flat without curvature both before and after deformation, and the 2D coordinates are defined by a simple projection along the direction perpendicular to the surface (e.g., $\boldsymbol{u} = (u^1, u^2) = (x^1, x^2)$), then both quantities have simpler forms as previously reported[5]. In these cases, area growth is given by $\gamma(p) = \det[\tilde{F}(H(p))]$, and the direction of deformation anisotropy is just given as the eigenvector of $\tilde{C}$ (i.e., $V^1 = \tilde{V}^1, V^2 = \tilde{V}^2$, $V^3 = 0$). The magnitude of anisotropy is the same as in the general case, $1 - \sqrt{\lambda_2 / \lambda_1}$ (or $\sqrt{\lambda_1 / \lambda_2}$).

It should be noted that the area growth rate and deformation anisotropy defined above are quantities defined in the configuration before deformation.

2-2. Relationship of eigenvalues/eigenvectors of deformation tensors between 2D and 3D representations.

As defined above, let C be the 3D representation of the right Cauchy-Green deformation tensor that is restricted on the tangent plane of the focal (curved) epithelial sheet, and λ and $\tilde{V}$ be the eigenvalues and eigenvectors of its 2D representation $\tilde{C}$. We now present proofs of the following:

(i) The eigenvalues of the deformation tensor $\tilde{C}^\alpha{}_\beta$ for 2D representations are also those of $C^i{}_j$ for 3D representations,

(ii) The push-forward of the eigenvectors of $\tilde{C}^{\alpha}{}_{\beta}$ gives those of $C^{i}{}_{j}$.

Note that in this subsection, we adopt Einstein summation convention.

Proof:

From the assumption $\tilde{C}^{\alpha}{}_{\beta}\tilde{V}^{\beta} = \lambda \tilde{V}^{\alpha}$, multiplying both sides by $\tilde{G}_{\delta\alpha}$, the following holds:

$$\tilde{C}_{\delta\beta}\tilde{V}^{\beta} = \lambda \tilde{G}_{\delta\alpha}\tilde{V}^{\alpha}. \tag{S10}$$

Using the following relationships shown before,

$$\tilde{G}_{\delta\alpha} = \delta_{ij}\left(\frac{\partial X^{i}}{\partial U^{\delta}}\right)\left(\frac{\partial X^{j}}{\partial U^{\alpha}}\right) \quad \text{(the definition of induced metric } \tilde{G}\text{)}, \tag{S3}$$

$$V^{i} = \left(\frac{\partial X^{i}}{\partial U^{\beta}}\right)\tilde{V}^{\beta} \quad \text{(the definition of } \boldsymbol{V} \text{ as the push-forward of } \tilde{V}\text{)}, \tag{S8}$$

$$\tilde{C}_{\alpha\beta} = C_{ij}\left(\frac{\partial X^{i}}{\partial U^{\alpha}}\right)\left(\frac{\partial X^{j}}{\partial U^{\beta}}\right), \tag{S9}$$

the left-hand and right-hand sides of Eq. (S10) are rewritten as follows:

$$\tilde{C}_{\delta\beta}\tilde{V}^{\beta} = C_{ij}\left(\frac{\partial X^{i}}{\partial U^{\delta}}\right)V^{j} = \delta_{im}C^{m}{}_{j}\left(\frac{\partial X^{i}}{\partial U^{\delta}}\right)V^{j} = \left\langle C^{m}{}_{j}V^{j}\mathbf{E}_{m}, \frac{\partial X^{i}}{\partial U^{\delta}}\mathbf{E}_{i}\right\rangle,$$

$$\lambda\tilde{G}_{\delta\alpha}\tilde{V}^{\alpha} = \lambda\delta_{ij}\left(\frac{\partial X^{i}}{\partial U^{\delta}}\right)\left(\frac{\partial X^{j}}{\partial U^{\alpha}}\right)\tilde{V}^{\alpha} = \lambda\delta_{ij}\left(\frac{\partial X^{i}}{\partial U^{\delta}}\right)V^{j} = \left\langle \lambda V^{j}\mathbf{E}_{j}, \frac{\partial X^{i}}{\partial U^{\delta}}\mathbf{E}_{i}\right\rangle,$$

where $\mathbf{E}_{\bullet}$ $(\bullet = 1,2,3)$ are orthonormal bases in 3D Euclidean space, and $\langle\, , \rangle$ is the inner product. Because both $C^{m}{}_{j}V^{j}\mathbf{E}_{m}$ and $\lambda V^{j}\mathbf{E}_{j}$ are 3D vectors on the tangent plane of the sheet, and because they return the same values of the inner product with two different vectors $\frac{\partial X^{i}}{\partial U^{\delta}}\mathbf{E}_{i}$ ($\delta = 1,2$), they must be equivalent. Consequently, the following relationship holds:

$$C^{m}{}_{j}V^{j} = \lambda V^{j},$$

that is, λ is also the eigenvalue of C. In addition, from the above, it is clear that the push-forward of $\tilde{V}$, i.e.

$V^i = \left(\frac{\partial X^i}{\partial U^\beta}\right)\tilde{V}^\beta$, is the eigenvector of C.

Supplementary Note 3: Bayesian reconstruction of tissue deformation maps from positional data of sparsely-labelled cells

In this section, we explain in detail the inference procedure for tissue deformation maps. The method proposed here is a generalization from our previous study where we focused on flat tissue deformation dynamics[5,6]. As shown in Fig. 1, organs and organoids often have complex morphologies with large curvature. Thus, when analyzing deformation dynamics of such bodies, the handling of curvilinear coordinate systems with non-Euclidean metrics is unavoidable. The biggest advantage of the new method we propose here is the applicability to arbitrary organs (or manifolds) represented with arbitrary coordinate systems and metrics, enabling us to overcome the difficulty in analyzing tissue deformation dynamics with complex morphology.

3-1. Discretization of tissue deformation map $\tilde{\phi}$

In our method, the tissue deformation map is represented as the deformation of a lattice for the given set of 2D coordinate systems (H and h); i.e., the correspondence between spatial coordinates of each lattice point before deformation (denoted by $\mathbf{\Theta}_\alpha = (U^1_{\Theta(\alpha)}, U^2_{\Theta(\alpha)})$ for α -th point, $\alpha = 1,\cdots,N_{Lattice}$) and after deformation ($\mathbf{\theta}_\alpha = (u^1_{\theta(\alpha)}, u^2_{\theta(\alpha)})$) (Supplementary Fig. 2). The lattice does not have to be regular, and the lattice interval may be different along each axis. Subsequently, the coordinates after deformation of an arbitrary point (especially an off-lattice point) on an epithelial sheet is modeled as follows. Suppose that the point is located at $\boldsymbol{U}_i = (U^1_i, U^2_i)$ before deformation and included in a square formed by the four lattice points i_1, i_2, i_3, and i_4 (Supplementary Fig. 2), and also suppose that position $\boldsymbol{U}_i$ is represented as a weighted linear sum of the coordinates of those four lattice points (denoted by $\mathbf{\Theta}_{i_k}$, $k = 1,\cdots,4$):

$$\boldsymbol{U}_i = (U^1_i, U^2_i) = \sum_k W_{i_k}\mathbf{\Theta}_{i_k} = W_{(i)}\mathbf{\Theta}_{nbd(i)}, \quad \text{(S11)}$$

$$W_{(i)} \equiv \begin{pmatrix} W_{i_1} & \cdots & W_{i_4} & 0 & \cdots & 0 \\ 0 & \cdots & 0 & W_{i_1} & \cdots & W_{i_4} \end{pmatrix},$$

$$\mathbf{\Theta}_{nbd(i)} \equiv \left(U^1_{\theta(i_1)},\cdots,U^1_{\theta(i_4)},U^2_{\theta(i_1)},\cdots,U^2_{\theta(i_4)}\right).$$

Then, the position after deformation of the focal point is modeled as the weighted linear sum of the coordinates

after deformation of the same four lattice points (denoted by $\boldsymbol{\theta}_{ik}$ $(k=1,\cdots,4)$) with the same weighting coefficients W_{i_k} as before deformation:

$$\boldsymbol{u}_i=(u_i^1,u_i^2)=\sum_k W_{i_k}\boldsymbol{\theta}_{i_k}=W_{(i)}\boldsymbol{\theta}_{nbd(i)}, \qquad \text{(S12)}$$

$$\boldsymbol{\theta}_{nbd(i)}\equiv\left(u^1_{\theta(i_1)},\cdots,u^1_{\theta(i_4)},u^2_{\theta(i_1)},\cdots,u^2_{\theta(i_4)}\right).$$

In the inference process, this relates data of the observed positions of labelled-cells with the positions after deformation of lattice points that are to be inferred. As interpolation coefficients W_{i_k} in Eqs. (S11) and (S12), we use the following shape functions adopted from our previous study[5]:

$$W_{ik}=\frac{1}{4}\left(1\pm\frac{dU^1{}_i}{\Delta_1}\right)\left(1\pm\frac{dU^2{}_i}{\Delta_2}\right),$$

where $d\boldsymbol{U}_i\equiv(dU_i^1,dU_i^2)=\boldsymbol{U}_i-\sum_k\boldsymbol{\Theta}_{ik}/4$. $\sum_k\boldsymbol{\Theta}_{ik}/4$ is the coordinate for the center of the square enveloping the focal point before deformation. Δ_1 and Δ_2 are the lattice intervals along the U^1-axis and U^2-axis, respectively.

3-2. A Bayesian statistical model: (i) data distribution

In our method, tissue deformation maps are estimated based on a Bayesian approach using positional data from randomly- and sparsely-labelled cells. Such a statistical approach is necessary because the positional data include different types of kinematic randomness, i.e., stochasticity in cell trajectories originating from the randomness of cell division orientation, the rearrangement of cell position through push-and-pull dynamics between neighboring cells, embryo-to-embryo variability in deformation dynamics, and measurement noise. A major advantage of the Bayesian approach is easier inclusion of plausible assumptions, such as deformation smoothness inside the tissue and at the boundary, into prior probability distributions[5,7].

In the main text, for clarity, we briefly summarized the key concepts of our modeling. Here, we explain the mathematical details of the modeling. In the estimation process, the posterior probability has the following form:

$$P(\boldsymbol{\theta}\,|\,\boldsymbol{u})\propto P(\boldsymbol{u}\,|\,\boldsymbol{\theta})\pi_1(\boldsymbol{\theta}\,|\,\boldsymbol{\eta}_1)\pi_2(\boldsymbol{\eta}_1\,|\,\boldsymbol{\eta}_2),$$

that is, our model includes three types of probability distributions: data distribution $P(\boldsymbol{u}\,|\,\boldsymbol{\theta})$, prior distributions of parameters $\pi_1(\boldsymbol{\theta}\,|\,\boldsymbol{\eta}_1)$, and of hyper-parameters $\pi_2(\boldsymbol{\eta}_1\,|\,\boldsymbol{\eta}_2)$. The 2D deformation map $\tilde{\phi}$ (more precisely, the map discretized by the lattice) is obtained by maximizing the marginal likelihood defined using those probability distributions. In this subsection, we explain the data distribution; the prior distributions and parameter estimation process will be explained in the next subsections.

What is estimated is the discretized 2D deformation map defined in previous subsection 3-1:

$$\boldsymbol{\theta} \equiv (u^1_{\theta(1)}, \cdots, u^1_{\theta(M_1)}, u^2_{\theta(1)}, \cdots, u^2_{\theta(M_1)}), \tag{S13}$$

where M_1 is the total number of lattice points. $\boldsymbol{\theta}$ acts as statistical parameters in the probability distribution for observing the data $\boldsymbol{u}_{data} \equiv (u^1_1, \cdots, u^1_N, u^2_1, \cdots, u^2_N)$ (N is the total number of labelled-cells). In the inference process, for given $\boldsymbol{u}_{data}$, this probability distribution gives the likelihood for the parameters $\boldsymbol{\theta}$ (also see subsection 3-4).

For modeling the data distribution, we start with the following assumptions: the level of noise is not so large compared to the curvature radius at each position on the surface after deformation, and for 3D representations (i.e., when the surface is embedded in 3D Euclidean space), the noise can be approximated as an additive and isotropic Gaussian distribution on the tangent plane at the focal position (Supplementary Fig. 2). Thus, the destination (i.e., the position after deformation) of the cell located at $\boldsymbol{X}$ before deformation is modeled as,

$$\boldsymbol{x} = \phi(\boldsymbol{X}) + \boldsymbol{\xi} \quad \text{or} \quad \begin{pmatrix} x^1 \\ x^2 \\ x^3 \end{pmatrix} = \begin{pmatrix} \phi^1(X^1, X^2, X^3) + \xi^1 \\ \phi^2(X^1, X^2, X^3) + \xi^2 \\ \phi^3(X^1, X^2, X^3) + \xi^3 \end{pmatrix}, \tag{S14}$$

where $\boldsymbol{\xi} \equiv (\xi^1, \xi^2, \xi^3)$ is the deviation obeying $N(\phi(\boldsymbol{X}), \Sigma)$. $(\Sigma)^{ij}$ is the variance-covariance matrix $(i, j = 1,2,3)$, and $(\Sigma^{-1})_{ij}$ is its inverse matrix whose components are given by $\frac{1}{\sigma^2}\delta_{ij}$. δ_{ij} and σ^2 are the Kronecker delta and the magnitude of noise, respectively. Note that the three components of noise ξ^i are not independent of each other because the deviation is on the tangent plane.

To estimate the 2D deformation map, the noise distribution of the 3D representation defined above needs to be transformed into a 2D version (Supplementary Fig. 2). The key point here is that, different from the 3D Euclidean situation, the noise distribution is not necessarily concentric but anisotropic with position-dependent anisotropy. This is due to distortion by the non-Euclidean metric tensor whose value can differ largely between the surface positions. It should be noted that the distortion also depends on the 2D coordinate chart adopted for describing the surface (Fig. 2A). This can be intuitively understood by considering the following simple case: the surface in 3D space is described as a graph $x^3 = f(x^1, x^2)$ and the chart (2D-coordinates) is given by a simple projection, i.e. $(u^1, u^2) = (x^1, x^2)$. In this case, the variance-covariance matrix of the noise distribution in (u^1, u^2)-space is given by

$$\Sigma_u = \sigma^2 \begin{pmatrix} 1+(\partial f/\partial u^1)^2 & (\partial f/\partial u^1)(\partial f/\partial u^2) \\ (\partial f/\partial u^1)(\partial f/\partial u^2) & 1+(\partial f/\partial u^2)^2 \end{pmatrix}^{-1}. \quad \text{(S15)}$$

Thus, the deviation from the isotropic distribution is larger at the point where the surface gradients $\partial f/\partial u^1$ and/or $\partial f/\partial u^2$ are steeper.

In the case of a general 2D-coordinate system (u^1, u^2), using the discretized map, the destination of a cell located at a given position before deformation $\boldsymbol{U}_i = (U_i^1, U_i^2)$ is given by:

$$\begin{pmatrix} u_i^1 \\ u_i^2 \end{pmatrix} = \begin{pmatrix} \tilde{\phi}^1(U_i^1, U_i^2) + \tilde{\xi}^1 \\ \tilde{\phi}^2(U_i^1, U_i^2) + \tilde{\xi}^2 \end{pmatrix} \cong \begin{pmatrix} (W_{(i)}\boldsymbol{\theta}_{nbd(i)})^1 + \tilde{\xi}^1 \\ (W_{(i)}\boldsymbol{\theta}_{nbd(i)})^2 + \tilde{\xi}^2 \end{pmatrix}, \quad \text{(S16)}$$

where the deviation $\tilde{\boldsymbol{\xi}} \equiv (\tilde{\xi}^1, \tilde{\xi}^2)$ obeys the following probability distribution:

$$P_{(i)}(\boldsymbol{u}_i \mid \boldsymbol{\theta}_{nbd(i)}, \boldsymbol{U}_i, \tilde{\Sigma}_{(i)}) = \frac{1}{2\pi \left|\Sigma_{(i)}\right|^{1/2}} \exp\left[-\frac{1}{2} (\boldsymbol{u}_i - W_{(i)}\boldsymbol{\theta}_{nbd(i)})^{\mathrm{T}} \tilde{\Sigma}_{(i)}^{-1} (\boldsymbol{u}_i - W_{(i)}\boldsymbol{\theta}_{nbd(i)}) \right]. \quad \text{(S17)}$$

Since the following relationship holds:

$$(\tilde{\Sigma}^{-1})_{\alpha\beta} = \sum_{i,j=1}^{3} (\Sigma^{-1})_{ij} \frac{\partial x^i}{\partial u^\alpha} \frac{\partial x^j}{\partial u^\beta} = \frac{1}{\sigma^2} \sum_{i,j=1}^{3} \delta_{ij} \frac{\partial x^i}{\partial u^\alpha} \frac{\partial x^j}{\partial u^\beta},$$

$$= \frac{1}{\sigma^2} \tilde{g}_{\alpha\beta}, \qquad (\alpha, \beta = 1,2)$$

$$\Leftrightarrow \tilde{\Sigma} = \sigma^2 \tilde{g}^{-1}, \quad \text{(S18)}$$

compared to the isotropic distribution, this distribution is shrunk by $1/\sqrt{\lambda_{i,1}}$ (or $1/\sqrt{\lambda_{i,2}}$) -fold along the $\boldsymbol{v}_{i,1}$ (or $\boldsymbol{v}_{i,2}$) direction, where $\lambda_{i,\bullet}$ and $\boldsymbol{v}_{i,\bullet}$ are the eigenvalue and its corresponding eigenvector of the induced metric $\tilde{g}$ (Eq. (S4)).

We need to be careful that, in the data distribution defined above, the variance-covariance matrix is calculated using the metric tensor at $\boldsymbol{u} = W_{(i)}\boldsymbol{\theta}_{nbd(i)}$. However, $\boldsymbol{\theta}_{nbd(i)}$ is the value that is to be inferred from the observed data, i.e., we do not know it a priori. Thus, in the practical inference process, the induced metric $\tilde{g}$ at the data point $\boldsymbol{u}_i$ after deformation that can be calculated a priori is used in place of the true value $\tilde{g}$ at

$W_{(i)}\boldsymbol{\theta}_{nbd(i)}$. Whether this replacement for approximation works well or not may depend on focal problems, but as we will show later, the answer is affirmative at least using our *in silico* and *in vivo* validation processes (Fig. 4). In what follows, the induced metric $\tilde{g}_{(i)}$ and the variance-covariance matrix $\tilde{\Sigma}_{(i)}$ represent their values calculated at the data point $\boldsymbol{u}_i$.

Before moving on to the next subsections, we rewrite Eq. (S17), which is necessary for the parameter estimation process in subsection 3-4. The exponent in Eq. (S17) can be rewritten as:

$$
\begin{aligned}
&-\frac{1}{2}(\boldsymbol{u}_i - W_{(i)}\boldsymbol{\theta}_{nbd(i)})^{\mathrm{T}}\tilde{\Sigma}_{(i)}^{-1}(\boldsymbol{u}_i - W_{(i)}\boldsymbol{\theta}_{nbd(i)}) \\
&= -\frac{1}{2\sigma^2}(\boldsymbol{u}_i - W_{(i)}\boldsymbol{\theta}_{nbd(i)})^{\mathrm{T}}\tilde{g}_{(i)}(\boldsymbol{u}_i - W_{(i)}\boldsymbol{\theta}_{nbd(i)}) \\
&= -\frac{1}{2\sigma^2}(\boldsymbol{u}_i - W_{(i)}\boldsymbol{\theta}_{nbd(i)})^{\mathrm{T}}(\tilde{g}_{(i)}^{1/2})^{T}\tilde{g}_{(i)}^{1/2}(\boldsymbol{u}_i - W_{(i)}\boldsymbol{\theta}_{nbd(i)}) \\
&= -\frac{1}{2\sigma^2}\left\|\tilde{g}_{(i)}^{1/2}(\boldsymbol{u}_i - W_{(i)}\boldsymbol{\theta}_{nbd(i)})\right\|^2, \qquad \text{(S19)}
\end{aligned}
$$

where $\tilde{g}_{(i)}^{1/2}$ is defined by $(\tilde{g}_{(i)}^{1/2})^{T}\tilde{g}_{(i)}^{1/2} = \tilde{g}_{(i)}^{1/2}\tilde{g}_{(i)}^{1/2} = \tilde{g}_{(i)}$, and its components are given as:

$$
\tilde{g}_{(i)}^{1/2} = \begin{pmatrix} \sqrt{\lambda_{i,1}} & 0 \\ 0 & \sqrt{\lambda_{i,2}} \end{pmatrix}\begin{pmatrix} v_{i,1,x} & v_{i,1,y} \\ -v_{i,1,y} & v_{i,1,x} \end{pmatrix} = \begin{pmatrix} \sqrt{\lambda_{i,1}}v_{i,1,x} & \sqrt{\lambda_{i,1}}v_{i,1,y} \\ -\sqrt{\lambda_{i,2}}v_{i,1,y} & \sqrt{\lambda_{i,2}}v_{i,1,x} \end{pmatrix}, \qquad \text{(S20)}
$$

where $\lambda_{i,\bullet}$ and $\boldsymbol{v}_{i,\bullet} = (v_{i,\bullet,x}, v_{i,\bullet,y})$ ($\bullet = 1,2$) are the eigenvalue and its corresponding unit eigenvector of the induced metric $\tilde{g}_{(i)}$, respectively. Using the following relationships:

$$
\sqrt{\det(\tilde{\Sigma}_{(i)})} = \sqrt{\det(\sigma^2\tilde{g}_{(i)}^{-1})} = \frac{\sigma^2}{\sqrt{\lambda_{i,1}\lambda_{i,2}}},
$$

$$
\boldsymbol{u}_i' \equiv \tilde{g}_{(i)}^{1/2}\boldsymbol{u}_i,
$$

$$
W'_{(i)} \equiv \tilde{g}_{(i)}^{1/2}W_{(i)} = \begin{pmatrix} (W'_{(i)})_{11} & \cdots & (W'_{(i)})_{18} \\ (W'_{(i)})_{21} & \cdots & (W'_{(i)})_{28} \end{pmatrix},
$$

Eq. (S17), the probability distribution for observing data point $\boldsymbol{u}_i$, is rewritten as:

$$P_{(i)}(\boldsymbol{u}_i \mid \boldsymbol{\theta}_{nbd(i)}, \boldsymbol{U}_i, \tilde{\Sigma}_{(i)}) = \frac{\sqrt{\lambda_{i,1}\lambda_{i,2}}}{2\pi\sigma^2} \exp\left[-\frac{1}{2\sigma^2} \left\| \boldsymbol{u}_i' - W'_{(i)} \boldsymbol{\theta}_{nbd(i)} \right\|^2 \right]. \qquad \text{(S17')}$$

In a similar way, for a given map $\boldsymbol{\theta} \equiv (u^1_{\theta(1)}, \cdots, u^1_{\theta(M_1)}, u^2_{\theta(1)}, \cdots, u^2_{\theta(M_1)})$, positional data for all labelled cells before deformation $\boldsymbol{U}_{data} \equiv (U^1_1, \cdots, U^1_N, U^2_1, \cdots, U^2_N)$, and a set of variance-covariance matrices defined at each data point after deformation $\tilde{\Sigma} \equiv \{\tilde{\Sigma}_{(1)}, \cdots, \tilde{\Sigma}_{(N)}\}$ (each component is defined as $\tilde{\Sigma}_{(i)} \equiv \sigma^2 \tilde{g}^{-1}_{(i)}$), the probability distribution for observing all the data $\boldsymbol{u}_{data} \equiv (u^1_1, \cdots, u^1_N, u^2_1, \cdots, u^2_N)$ is modeled as:

$$P(\boldsymbol{u}_{data} \mid \boldsymbol{\theta}, \boldsymbol{U}_{data}, \tilde{\Sigma})$$

$$= \prod_{i=1}^{N} \frac{1}{2\pi |\tilde{\Sigma}_{(i)}|^{1/2}} \exp\left[-\frac{1}{2} (\boldsymbol{u}_i - W_{(i)} \boldsymbol{\theta}_{nbd(i)})^{\mathrm{T}} \tilde{\Sigma}^{-1}_{(i)} (\boldsymbol{u}_i - W_{(i)} \boldsymbol{\theta}_{nbd(i)}) \right],$$

$$= \frac{\prod_{i=1}^{N} \sqrt{\lambda_{i,1}\lambda_{i,2}}}{(2\pi\sigma^2)^N} \exp\left[-\frac{1}{2\sigma^2} \left\| \boldsymbol{u}' - W' \boldsymbol{\theta} \right\|^2 \right], \qquad \text{(S21)}$$

where $\boldsymbol{u}' \equiv (u'^1_1, \cdots, u'^1_N, u'^2_1, \cdots, u'^2_N)$ (see Supplementary Fig. 4 for the definition of matrix W').

3-3. Prior distributions: smooth deformation of internal tissues and tissue boundary

As shown in our previous study[5], the assumption of smooth deformation of internal tissues and organ boundaries as Bayesian prior information worked well for inferring the deformation maps; this prevents the overfitting to noise in the data and divergence at the boundary (i.e., having extremely large numbers). Here we adopt the same assumptions and briefly summarize them.

Let us consider the deformation around each lattice point $\boldsymbol{\Theta}_\alpha$. It can be approximated as the deformation of four squares formed with adjacent lattice points of $\boldsymbol{\Theta}_\alpha$, $\boldsymbol{\Theta}_{\alpha,1}$, $\boldsymbol{\Theta}_{\alpha,2}$, $\boldsymbol{\Theta}_{\alpha,3}$, and $\boldsymbol{\Theta}_{\alpha,4}$ (Supplementary Fig. 2). For example, the square spanned by the two vectors $\boldsymbol{\Theta}_{\alpha,1} - \boldsymbol{\Theta}_\alpha$ and $\boldsymbol{\Theta}_{\alpha,2} - \boldsymbol{\Theta}_\alpha$ deforms into the parallelogram spanned by $\boldsymbol{\theta}_{\alpha,1} - \boldsymbol{\theta}_\alpha$ and $\boldsymbol{\theta}_{\alpha,2} - \boldsymbol{\theta}_\alpha$, which defines the linear transformation or deformation gradient tensor, $\tilde{\boldsymbol{F}}^{(\alpha,1)}$. As a representation of smooth tissue deformation, we adopted the following model: the expectation of $\boldsymbol{\theta}_\alpha$ is chosen so as to minimize the variance of the four deformation

gradient tensors around that point:

$$\mu_{\theta_\alpha} = \arg\min \sum_{j=1}^{4} \sum_{k,L} \left(\left(\tilde{F}^{(\alpha,j)}\right)^k{}_L - E\left[\left(\tilde{F}^{(\alpha)}\right)^k{}_L\right] \right)^2 . \qquad \text{(S22)}$$

Intuitively, this means that the difference among the deformations of the four squares is not large. After some calculations, Eq. (S22) takes on the following form with constant matrix $B_{\alpha,j}$:

$$\mu_{\theta_\alpha} = \sum_{j=1}^{4} B_{\alpha,j} \boldsymbol{\theta}_{\alpha,j} . \qquad \text{(S23)}$$

Next, the prior distribution for $\boldsymbol{\theta}_\alpha$ is given by the Gaussian distribution around this expectation with the variance w_1^2 (as will be shown later, w_1^2 is the quantity to be estimated):

$$N(\mu_{\theta_\alpha}, w_1^2) = \frac{1}{\sqrt{2\pi w_1^2}} \exp\left[-\frac{1}{2w_1^2} \left\| \boldsymbol{\theta}_\alpha - \sum_{j=1}^{4} B_{\alpha,j} \boldsymbol{\theta}_{\alpha,j} \right\|^2 \right]. \qquad \text{(S24)}$$

Note that if $\boldsymbol{\theta}_\alpha$ is on the edge of the calculation domain, Eq. (S24) needs to be slightly modified using the hyper-parameters $\boldsymbol{\eta}_{1,\beta}$ representing the position of the boundary points (Supplementary Fig. 2). Finally, by multiplying the prior distributions for all the lattice points, the prior distribution for $\boldsymbol{\theta} \equiv (u^1_{\theta(1)}, \cdots, u^1_{\theta(M_1)}, u^2_{\theta(1)}, \cdots, u^2_{\theta(M_1)})$ becomes:

$$\pi_1(\boldsymbol{\theta} \mid w_1^2, \boldsymbol{\eta}_1) = \frac{(\det D_1^T D_1)^{1/2}}{(2\pi w_1^2)^{M_1}} \exp\left[-\frac{1}{2w_1^2} \left\| D_1 \boldsymbol{\theta} - B_1 \boldsymbol{\eta}_1 \right\|^2 \right], \qquad \text{(S25)}$$

where the matrices D_1 and B_1 are calculated based on Eq. (S24).

Similar to the deformation internal to the tissues, a smooth deformation of the boundary is assumed. This is modeled for each boundary point $\boldsymbol{\eta}_{1,\beta}$ as:

$$E[\boldsymbol{\eta}_{1,\beta}] = (\boldsymbol{\eta}_{1,\beta-1} + \boldsymbol{\eta}_{1,\beta+1})/2 \qquad \text{(S26)}$$

$$Var[\boldsymbol{\eta}_{1,\beta}] = (w_2)^2 \left(\left\| \boldsymbol{\eta}_{1,\beta+1} - \boldsymbol{\eta}_{1,\beta} \right\| + \left\| \boldsymbol{\eta}_{1,\beta} - \boldsymbol{\eta}_{1,\beta-1} \right\| \right)/2 . \qquad \text{(S27)}$$

Together, the prior information for all the hyper-parameters is given by the following Gaussian distribution:

$$\pi_2(\boldsymbol{\eta}_1 \mid w_2^2, \boldsymbol{\eta}_2) = \frac{(\det D_2^T D_2)^{1/2}}{(2\pi w_2^2)^{M_2}} \exp\left[-\frac{1}{2w_2^2} \left\| D_2 \boldsymbol{\eta}_1 - B_2 \boldsymbol{\eta}_2 \right\|^2 \right], \qquad \text{(S28)}$$

where $\boldsymbol{\eta}_2$ is another hyper-parameter (Supplementary Fig. 3).

3-4. Parameter estimation

Now that all three probability distributions have been clarified, the posterior probability is given as follows:

$$P(\boldsymbol{\theta} \mid \boldsymbol{u}_{data}, \boldsymbol{U}_{data}, \tilde{\Sigma}) \propto P(\boldsymbol{u}_{data} \mid \boldsymbol{\theta}, \boldsymbol{U}_{data}, \tilde{\Sigma}) \pi_1(\boldsymbol{\theta} \mid w_1^{\,2}, \boldsymbol{\eta}_1) \pi_2(\boldsymbol{\eta}_1 \mid w_2^{\,2}, \boldsymbol{\eta}_2),$$

$$\propto \exp\left[-\frac{1}{2\sigma^2} \left(\|\boldsymbol{u}' - W'\boldsymbol{\theta}\|^2 + \mu_1^{\,2} \|D_1\boldsymbol{\theta} - B_1\boldsymbol{\eta}_1\|^2 + \mu_2^{\,2} \|D_2\boldsymbol{\eta}_1 - B_2\boldsymbol{\eta}_2\|^2 \right) \right], \quad \text{(S29)}$$

where $\mu_1 \equiv \sigma^2 / w_1^{\,2}$ and $\mu_2 \equiv \sigma^2 / w_2^{\,2}$. The hyper-parameters $\{\sigma^2, \mu_1^{\,2}, \mu_2^{\,2}, \boldsymbol{\eta}_2\}$ are determined by maximizing the logarithm of the marginal likelihood function $\log L(\sigma^2, \mu_1^{\,2}, \mu_2^{\,2}, \boldsymbol{\eta}_2)$ defined as follows[5,8,9]:

$$\log L(\sigma^2, \mu_1^{\,2}, \mu_2^{\,2}, \boldsymbol{\eta}_2)$$

$$= \log \iint P(\boldsymbol{u}_{data} \mid \boldsymbol{\theta}, \boldsymbol{U}_{data}, \tilde{\Sigma}) \pi_1(\boldsymbol{\theta} \mid w_1^{\,2}, \boldsymbol{\eta}_1) \pi_2(\boldsymbol{\eta}_1 \mid w_2^{\,2}, \boldsymbol{\eta}_2) d\boldsymbol{\theta} d\boldsymbol{\eta}_1. \quad \text{(S30)}$$

According to the calculation procedure in our previous study [see Appendices in [ref 5]], the problem ultimately comes down to numerically maximizing the function in regard to two hyper-parameters μ_1, μ_2. The most probable estimation for $\boldsymbol{\theta}$ is then obtained by maximizing the posterior probability given by Eq. (S29).

Supplementary Note 4: Validation of the proposed method using artificial data set

We evaluated the performance of our method using artificially-generated data (Figs. 4 and 5). As stated in the main text, considering the biological application to optic vesicle evagination from the neural tube, we generated test data from two types of tissue evagination models. Here, we explain the details of data generation and analyses in those models.

4-1. Deformation of a surface represented as a graph

We first considered a surface deformation in which the shape of the surface both prior to and after deformation can be represented as graphs, and the 2D coordinate system on the surface was obtained by simple projections (e.g., $(X, Y, Z) \mapsto (X, Y)$ in the following example). In this example, we specified the correspondence of positions before and after deformation for all points on the surface, in other words, the answer of deformation map ϕ was given. Thus, the performance of the proposed method can be directly evaluated by calculating the error between the estimated map and the answer of the map. The primary purpose of the analysis using this data was to test how the estimation performance was affected by the metric-dependent noise anisotropy as well as to confirm how well the proposed method works.

4-1-1. Defining the surface and deformation map

We considered a surface whose shape before and after deformation can be represented by the following graphs:

Before deformation: $Z = f^{before}(X,Y) = -c_1(X^2 + Y^2)$, (S31a)

After deformation: $Z = f^{after}(X,Y) = -c_1(X^2 + Y^2) + c_2 \exp(-(X^2 + Y^2)/c_3)$, (S31b)

where $(c_1, c_2, c_3) = (0.05, 10, 20)$. For simplicity, we used the common 3D Cartesian coordinate system before and after deformation defined as $(X,Y,Z) = (X^1, X^2, X^3) = (x^1, x^2, x^3)$. The 3D deformation map ϕ was then defined in the following manner: each point (X_0, Y_0, Z_0) on the surface before deformation was mapped to the intersection on the surface after deformation and the line through (X_0, Y_0, Z_0) and $(0,0,-c_2)$ given by:

$$(X,Y,Z) = (X_0, Y_0, Z_0 + c_2)t + (0,0,-c_2), \tag{S32}$$

where t is a parameter defining the line. The value of t at the intersection can be calculated by substituting the relationship Eq. (S32) and $Z_0 = f^{before}(X_0, Y_0)$ into $Z = f^{after}(X,Y)$. After some calculations, t at the intersection satisfies the following equation:

$$f(t) \equiv \frac{X_0^{\ 2} + Y_0^{\ 2}}{c_3} t^2 + \log(t-1) + \log\left(\frac{c_1}{c_2}(X_0^{\ 2} + Y_0^{\ 2})t + 1\right) = 0. \tag{S33}$$

Using the Newton method, we numerically solved Eq. (S33) from the recurrence formula:

$$t_{n+1} = t_n - f(t_n)/f'(t_n). \tag{S34}$$

4-1-2. Random labelling of cells on the surface

In order to generate test data, it is necessary to perform random sampling at the surface (corresponding to the random labelling of cells on the given surface). We must be careful in the computation of this sampling process as uniform random sampling from the 2D U^1-U^2 plane does not give uniform sampling from the target surface. This is because the metric tensor $\tilde{G}(\boldsymbol{U})$ is different depending on the position on the surface. Since the 2D coordinate chart $\boldsymbol{U} = H(\boldsymbol{X})$ is a generally nonlinear map, each portion of the surface is compressed into the 2D plane with some distortion. Specifically, for a given 2D coordinate system, the area around each point on the surface is multiplied by $1/\sqrt{\det \tilde{G}(\boldsymbol{U})}$. Therefore, to achieve uniform random sampling from the 2D U^1-U^2 plane, each point needs to be sampled with the weight $\sqrt{\det \tilde{G}(\boldsymbol{U})}$. In this study, we numerically approximated this using the following procedure:

(i) Before sampling, the U^1-U^2 plane was discretized by a fine mesh.
(ii) For each square element, its sampling weight was calculated using the metric tensor at the center of the element.
(iii) In each sampling step, first, one square element was randomly chosen with the weight defined above.
(iv) Then, a point inside the element was sampled using a uniform random number.

Steps (iii) and (iv) were repeated until the desired number of data points $\boldsymbol{U}_i$ $(i = 1, \cdots, N)$ was obtained.

4-1-3. Adding noise

For each sampled data point $\boldsymbol{U}_i$, using Eqs. (S31) and (S32), the corresponding 3D coordinates before and after deformation, $\boldsymbol{X}_i$ and $\boldsymbol{x}_i^0$ were calculated (0 indicates the position after deformation without noise). To mimic the assumed noise in cellular position in the actual biological data, we added noise to the position after deformation $\boldsymbol{x}_i^0$ as follows:

$$\boldsymbol{x}_i = \boldsymbol{x}_i^0 + \boldsymbol{\xi}_i,$$

where $\boldsymbol{x}_i$ is the 3D position after deformation of *i*-th cell with noise. The noise $\boldsymbol{\xi}_i$ obeys the isotropic Gaussian restricted on the tangent plane at $\boldsymbol{x}_i^0$ with the mean 0 and the variance σ^2. In the analysis with these data, we used two values for σ, $\sigma = 0.2$ and $\sigma = 1.0$; the former (or latter) corresponds to 10% (or 50%) of the mesh size in the estimation of deformation maps.

Then, using a simple projection, its correspondent 2D coordinate $\boldsymbol{u}_i$ was obtained. With the above procedure, N (=100 or 200) sets of 2D coordinates for randomly labelled cells before and after deformation were prepared.

4-1-4. Evaluation of estimation performance

We estimated maps using the data generated above and compared them with the true maps (Fig. 4). The estimation accuracy of each map was quantified by calculating the mean square error of the estimated positions of the lattice points after deformation for each data set:

$$\sqrt{\left(\sum_{i=1}^{N_L} \left\| \boldsymbol{\theta}_i - \hat{\boldsymbol{\theta}}_i \right\|^2\right) / N_L}\,, \qquad \text{(S35)}$$

where $\boldsymbol{\theta}_i$ and $\hat{\boldsymbol{\theta}}_i$ are the positional vectors of the *i*-th lattice points of the true and estimated maps,

respectively, and N_L is the total number of lattice points ($N_L = 121$). For noise with $\sigma = 0.2$ (or $\sigma = 1.0$), the estimation error relative to the mesh size was around 10% (or 25%); for reference, the prediction errors relative to the mesh size were about 20-30% in the data analysis for chick forebrain morphogenesis.

As stated in the main text, the primary conclusion from this analysis is that, when the magnitude of noise is not negligible, reflecting metric-dependent noise anisotropy in the estimation process is necessary for preventing bias error, i.e., the strong influence of estimation error on position. Since the biological data often include different types of noise, this is an important issue in the analysis of the actual data. In addition, although the accuracy of the estimation depends on multiple parameters such as the quantity of labelled cells and the noise intensity, sufficient accuracy is expected when the number of data points is comparable to the number of lattice points and the noise intensity is not large compared to the lattice size.

4-2. Vesicle deformation generated by mechanical simulation of an elastic membrane

The other set of validation data was generated by mechanically simulating evagination from a vesicle composed of elastic membrane, which more realistically reflects epithelial morphogenesis. Specifically, we tested two candidate models for tissue evagination: biased tissue growth through cell division as seen in plant root development, and mechanical anisotropy (whereby intercellular edges are easier to shrink in a specific direction) such as the mechanism of Drosophila germband extension (Fig. 5). In the latter model, cell division was not implemented. Here we explain the details of this modeling and its analysis.

4-2-1. Equation of motion and potential energy

As shown in Supplementary Fig. 5(A), each cell was modeled as a particle represented by its center position in 3D space, $\boldsymbol{x}_i$. The potential energy U was defined between adjacent cells. Through the energy, the cells mechanically interact with each other. The adjacent relationship among cells was determined by triangulation, and overall tissue morphology was represented by the network among interacting cells. From the duality between the triangulation and Voronoi tessellation, two ways of representation are possible (Supplementary Fig. 5(B)). The dynamics for the motion of each cell center was given by the following overdamped Langevin equation:

$$\eta \frac{\partial \boldsymbol{x}_i}{\partial t} = -\frac{\partial U}{\partial \boldsymbol{x}_i} + \xi_i(t)\boldsymbol{t}_i \;, \qquad \text{(S36)}$$

where η is the friction coefficient. The second term on the right hand side is the noise term. ξ_i is the white noise satisfying $E[\xi_i(t)] = 0$ and $E[\xi_i(t_1)\xi_j(t_2)] = s^2\delta_{ij}\delta(t_1 - t_2)$ with the noise intensity s. $\boldsymbol{t}_i$ is a unit tangent vector of the tissue surface with random orientation. The tangent vector was calculated based on the normal vector at the position located at the cell center defined as the average of the normal vectors of adjacent triangular elements (Supplementary Fig. 5(D)). The potential energy U was given as follows:

$$U = \frac{1}{2}\sum_{(i,j)}^{(cell-center)} \lambda_{ij}\left(l_{ij} - (r_i + r_j)\right)^2 + \frac{1}{2}M_{bend}\sum_{(\alpha,\beta)}^{(triangle)}\left(\mathbf{n}_\alpha - \mathbf{n}_\beta\right)^2 \qquad \text{(S37)}$$

where l_{ij} is the distance between the adjacent cells i and j, and r_i and r_i are their natural sizes, respectively. λ_{ij} is the coefficient for the tension between cells i and j. M_{bend} is the bending rigidity of the surface, and $\mathbf{n}_\alpha$ and $\mathbf{n}_\beta$ are the unit normal vectors of the triangles α and β adjacent to one another. This energy converges to that of a finite deformation of a thin plate when the size of the triangle mesh tends to zero[10].

As a result of cell movement and/or cell division, the neighboring relationship among cells (i.e., cell-cell network topology) can change. This was implemented by a process called remesh (Supplementary Fig. 5(F)), which is a dual representation of the T1 process (cell-cell rearrangement) in the vertex dynamics model used for the dynamics of epithelial tissues and foams[11, 12].

4-2-2. Scenario (I): Evagination induced by biased growth

As one possibility for achieving tissue evagination, we first assumed biased tissue growth. To accomplish this, a point source of morphogen was placed at a specific location on the initial vesicle, and another source was placed at its opposite side. The morphogen was then secreted from these sources to form a gradient. The concentration gradient was calculated by solving a discretized diffusion equation on the triangular mesh. The cell division rate was given by a step function of the concentration; i.e., only the cells exposed to a concentration beyond the threshold can divide. Each cell had a cell cycle. For simplicity, the cycle was composed of periods of non-growth (corresponding to the G1 phase) and cell growth (S or G2 phases). Cell division occurs instantaneously (i.e., M phase was neglected). In the period of cell growth, the growth process was implemented by gradually changing the natural size of each cell r over time as follows:

$$\tau_{S-G2}\dot{r} = cr_0 - r\,,$$

where τ_{S-G2} is the time constant. The orientation of cell division was randomly chosen on the tangent plane of the tissue surface. The initial number of cells was set to 1000, and cell division continued until the total number of cells reached 1200.

4-2-3. Scenario (II): Evagination induced by mechanical anisotropy

As another mechanism for achieving tissue evagination, we developed a model with tissue mechanical anisotropy, such that the tension/adhesion on each cell-cell edge had a directional dependence. Although there are multiple ways to achieve such anisotropy, we adopted a direction-dependent asymmetry in elastic energy, which was implemented in the following way. In the energy function given by Eq. (S37), for each edge, depending on the angle α with a specific axis (tissue evagination occurs in the direction of this axis), the value

of λ_{ij} was defined by:

$$\lambda_{ij} = \begin{cases} \lambda_1 + \lambda_2 \left|\cos\alpha\right| & l_{ij} < r_i + r_j \\ \lambda_1 + \lambda_2 \left(1 - \left|\cos\alpha\right|\right) & l_{ij} > r_i + r_j \end{cases}.$$

This function can be interpreted as follows. For simplicity, let us consider two extreme cases, (i) the direction of the focal edge is parallel to the evagination axis ($\alpha = 0$) and (ii) it is perpendicular to the axis ($\alpha = \pi/2$). When the edge length is shorter than the natural size $r_i + r_j$, the elastic restoring force on the vertical edge ($\lambda_{ij} = \lambda_1$) is weaker than that on the parallel one ($\lambda_{ij} = \lambda_1 + \lambda_2$). Oppositely, when the edge length is longer than the natural size, the force on the vertical edge is stronger. This asymmetrically-shaped energy function increases the chance that the vertical edge shrinks, although the length at equilibrium is the same for all edges.

4-2-4. Deformation analysis and evaluation of estimation performance

The 2D coordinates on the surface before and after deformation were defined using the spherical harmonics expansion (note that the mechanical simulations were performed in the 3D coordinate system). Since the cell centers were not necessarily present on the approximated surface, the 2D coordinate for each cell center was obtained by the projection with the minimum distance onto the approximated surface (the distance was very small compared to the natural length between adjacent cells). The total number of cells before deformation was 1000. We estimated 2D deformation maps for both scenarios (I: biased growth, II: mechanical anisotropy) using positional data from $N = 100$ or $N = 200$ cells. In the estimation processes, taking the morphological symmetry into consideration, we estimated the map for the region that includes one protrusion. The number of lattice points for approximating the map was $M = 105$. For Scenario (I), when a cell divides, one of its daughter cells is randomly chosen to be tracked.

In the validation process using the data generated by mechanical simulation, we did not have the answer of map ϕ, different from the previous case (see Supplementary Note 4-1). Therefore, we evaluated the accuracy of the estimated maps by measuring their predictive performance (cross-validation). The data of cell position before and after deformation for some cells was used to estimate the deformation maps in the proposed method, and the remaining data were used to calculate predictability, defined as the mean square error of the predicted positions of the cells after deformation. The sizes of the prediction errors were equivalent to about 1-2 cells, demonstrating good performance. The heat maps and statistics shown in Fig. 5 were calculated from the results of $N = 200$. In the calculation of the heat maps, we used finer meshes for which deformation characteristics were calculated by interpolation using the shape function W_{ik} (see Supplementary Note 3-1). Although the prediction error increases with a decrease in the number of data points used for the estimation, the errors in cases of both $N = 100$ and $N = 200$ were small enough compared to the lattice interval, demonstrating that the performance was acceptable when the number of data points was comparable to the

number of the lattice points, as in 4-1.

4-2-5. Parameter values

The values for the parameters used in the numerical simulations are as follows: $\eta = 1.0$, $\sigma = 0.6$, $r_0 = 1.0$, $\tau_{G1} = \tau_{S-G2} = 50.0$, $M = 1.5$, $\lambda_0 = 2.0$, $\lambda_1 = 1.0$, $\lambda_2 = 2.0$, $c = \sqrt{2}$.

Supplementary references

1. Holzapfel, G. A. Nonlinear Solid Mechanics, John Wiley & Sons, LTD, (2000).
2. Marseden, J. E. & Hughes, T. J. R. Mathematical foundations of elasticity, Dover publications, INC. New York, (1983).
3. Shen, L., Farid, H. & McPeek, M. A. Modeling three-dimentional morphological structures using spherical harmonics. *Evolution* 63, 1003–1016 (2009).
4. Blanchard, G. B., Murugesu, S., Adams, R. J., Martinez-Arias, A. & Gorfinkiel, N. Cytoskeletal dynamics and supracellular organisation of cell shape fluctuations during dorsal closure. *Development* 137, 2743–2752 (2010).
5. Morishita, Y. & Suzuki, T. Bayesian inference of whole-organ deformation dynamics from limited space-time point data. *Journal of Theoretical Biology* 357, 74–85 (2014).
6. Morishita, Y., Kuroiwa, A. & Suzuki, T. Quantitative analysis of tissue deformation dynamics reveals three characteristic growth modes and globally aligned anisotropic tissue deformation during chick limb development. *Development* 142, 1672–1683 (2015).
7. Bishop, C. M. Pattern recognition and machine learning. Springer, New York (2007).
8. Akaike, H. Likelihood and the Bayes procedure. In: Bernardo, J.M., DeGroot, M.H., Lindley, D.V., Smith, A.F.M. (Eds.), Bayesian Statistics. University Press, Valencia (In). (1980).
9. Ishihara, S. & Sugimura, K. Bayesian inference of force dynamics during morphogenesis. *Journal of Theoretical Biology* 313, 201-211 (2012).
10. Seung, H. S. & Nelson, D. R. Defects in flexible membranes with crystalline order. *Phys Rev A Gen Phys* 38, 1005–1018 (1988).
11. Nagai, T. & Honda, H. A dynamic cell model for the formation of epithelial tissues. *Philos. Mag. B* 81, 699-719 (2001).
12. Farhadifar, R., Röper, J.-C., Aigouy, B., Eaton, S. & Jülicher, F. The influence of cell mechanics, cell-cell interactions, and proliferation on epithelial packing. *Current Biology* 17, 2095–2104 (2007).
